# Viral Evasion of a Bacterial Suicide System by RNA–Based Molecular Mimicry Enables Infectious Altruism

**DOI:** 10.1371/journal.pgen.1003023

**Published:** 2012-10-18

**Authors:** Tim R. Blower, Terry J. Evans, Rita Przybilski, Peter C. Fineran, George P. C. Salmond

**Affiliations:** 1 Department of Biochemistry, University of Cambridge, Cambridge, United Kingdom; 2 Department of Microbiology and Immunology, University of Otago, Dunedin, New Zealand; Universidad de Sevilla, Spain

## Abstract

Abortive infection, during which an infected bacterial cell commits altruistic suicide to destroy the replicating bacteriophage and protect the clonal population, can be mediated by toxin-antitoxin systems such as the Type III protein–RNA toxin-antitoxin system, ToxIN. A flagellum-dependent bacteriophage of the *Myoviridae*, ΦTE, evolved rare mutants that “escaped” ToxIN-mediated abortive infection within *Pectobacterium atrosepticum*. Wild-type ΦTE encoded a short sequence similar to the repetitive nucleotide sequence of the RNA antitoxin, ToxI, from ToxIN. The ΦTE escape mutants had expanded the number of these “pseudo-ToxI” genetic repeats and, in one case, an escape phage had “hijacked” ToxI from the plasmid-borne *toxIN* locus, through recombination. Expression of the pseudo-ToxI repeats during ΦTE infection allowed the phage to replicate, unaffected by ToxIN, through RNA–based molecular mimicry. This is the first example of a non-coding RNA encoded by a phage that evolves by selective expansion and recombination to enable viral suppression of a defensive bacterial suicide system. Furthermore, the ΦTE escape phages had evolved enhanced capacity to transduce replicons expressing ToxIN, demonstrating virus-mediated horizontal transfer of genetic altruism.

## Author Summary

Bacteria are under constant attack by their viral parasites, bacteriophages, which outnumber bacteria by an estimated ten-to-one. The constant selection pressure from this predation promotes the evolution and dissemination of bacterial bacteriophage-resistance mechanisms. One family of protective systems causes the infected cell to undergo premature suicide, in an altruistic move that protects the clonal population of bacteria by blocking virus replication. We identified a means by which a bacteriophage counter-evolved to avoid one such system. This system relies on two components: a toxic part to kill the cell and an antidote that holds the toxin in check until required. The bacteriophage evolved sequences encoding mimics of the cellular antidote and expressed these mimics so that it could continue replicating without becoming a victim of the host's defensive system. Furthermore, this evolved bacteriophage was able to transfer the DNA encoding the defence system to a new bacterial host. In so doing, the evolved bacteriophage may have indirectly created populations of host cells inside which it could productively replicate, while also providing the host better protection from competing predators.

## Introduction

Toxin-antitoxin (TA) systems are ubiquitously distributed in plasmids and chromosomes of prokaryotes [1]–[3]. TA systems are divided into three Types, depending upon the nature of the interacting partners [4]. These small, bicistronic, genetic loci were originally identified as plasmid maintenance systems [5] though they are also involved in stress responses [6] and formation of persister cells [7] amongst other roles [8]. All three Types have been shown to protect from bacteriophage (phage) infection. The Type I *hok/sok* locus excludes T4 [9], the Type II *mazEF* locus can prevent spread of phage P1 infection [10] and the Type III *toxIN* locus can inhibit multiple phages in multiple host backgrounds [11], [12].

ToxIN was the first characterised Type III protein-RNA TA system, encoded by plasmid pECA1039 of the phytopathogen *Pectobacterium atrosepticum* [11]. Recent crystallographic studies showed that ToxN is a 19.7 kDa endoribonuclease of the Kid family, which is held inactive as a hetero-hexameric triangular structure, with three ToxN monomers in complex with three, 36-nucleotide, pseudoknots of antitoxic ToxI RNA [4], [13]. ToxIN was originally discovered through shared sequence similarity with an abortive infection (Abi) system, AbiQ, from *Lactococcus lactis* [14].

Due to constant selection pressure from viral predation, bacteria have developed multiple routes of defence; these include altering the cell surface to avoid phage adsorption, cleavage of viral nucleic acids by restriction-modification or CRISPR/Cas systems, preventing viral DNA injection, and through Abi systems [15]. Abi systems are lethal to their hosts when activated in a phage-infected cell. This prevents productive phage propagation – and thus protects the clonal bacterial population through ‘altruistic’ suicide [16]. By sequencing mutants of phages that had spontaneously ‘escaped’ an Abi system, it has been possible to examine the activity of the Abi systems and also the components of each phage required to activate these systems [17], [18].

To investigate how phages interact with the ToxIN dual functions (Abi and TA), we sought to characterise phage mutants resistant to ToxIN. A ToxIN-sensitive phage, ΦTE, which infects *Pectobacterium atrosepticum* 1043 (Pba) [19], evolved low-frequency spontaneous escape mutants that were ToxIN-insensitive. In this study, we have sequenced the genomes of both wild type and escape ΦTE phages in order to identify the factors involved in phage-ToxIN recognition.

## Results

### ΦTE is a flagellum-dependent rv5-like virus

**Figure 1:**
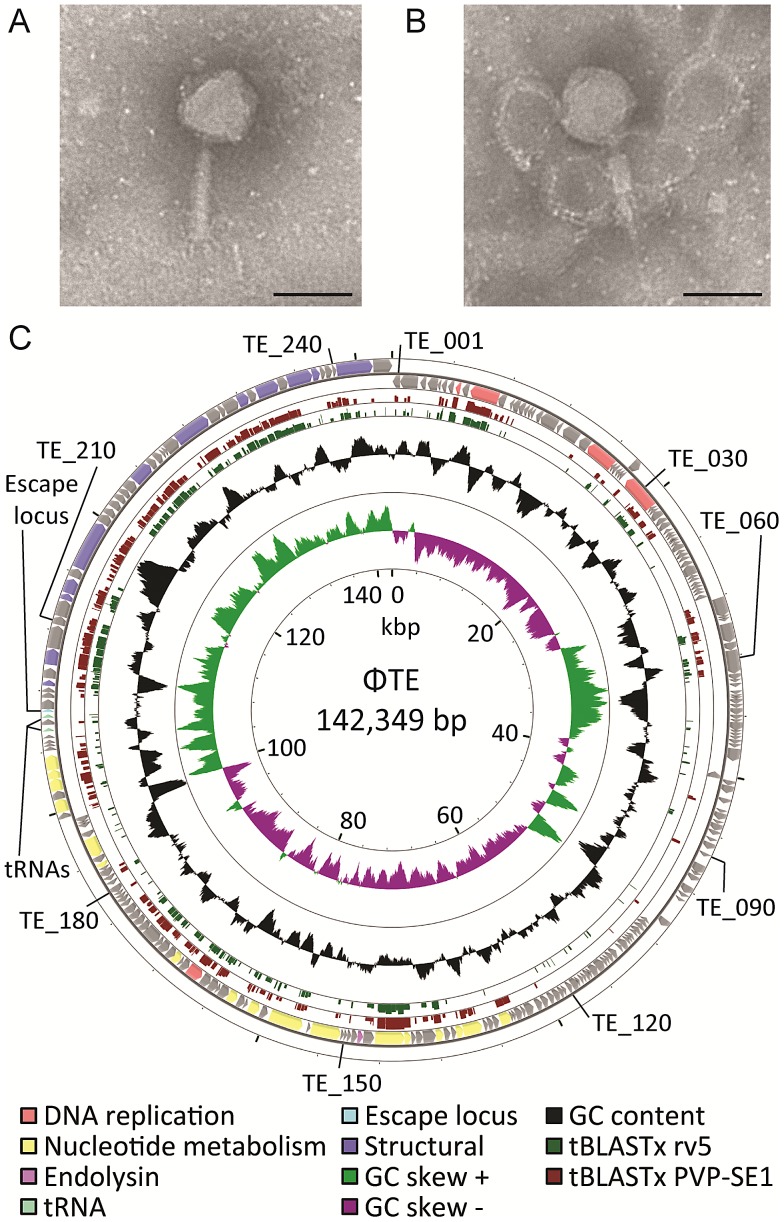
ΦTE morphology and genome overview. (A–B) Transmission electron micrographs of individual ΦTE virus particles. The tail is fully extended in (A) and contracted in (B). Each scale bar represents 100 nm. (C) Summary of the 142,349 bp circularly-permuted genome of ΦTE, including all ORFs (colour coded to function where possible), two tRNAs and the ncRNA comprising pseudo-ToxI, encoded by the escape locus (Table S2). Selected ΦTE genes are indicated by “TE_x” around the genome, for orientation. GC skew and GC content are shown along with the tBLASTx results against two related phages, coliphage rv5 and *Salmonella* phage PVP-SE1.

Mutant Pba strain SCC34 has a mucoid morphology that provides resistance to infection by lipopolysaccharide (LPS)-dependent phages, such as the generalised transducing phage ΦM1 [20], [21]. ΦTE was initially isolated from treated sewage effluent enriched for phages using SCC34 as host, as part of a screen for lipopolysaccharide (LPS)-independent phages. Using transmission electron microscopy, ΦTE was seen to have an isometric, icosahedral, head measuring 98 ±4 nm from flat face to flat face and a tail measuring 124±3 nm when extended (Figure 1A), or 65±7 nm when contracted (Figure 1B). ΦTE was classified as a member of the *Myoviridae* of the order Caudovirales [22]. A bank of ΦTE-resistant transposon-mutants of Pba was generated in order to identify the host receptor for ΦTE. These mutants of Pba remained sensitive to ΦM1 but were resistant to the flagellum-dependent phage, ΦAT1 [23]. Seven of the transposon insertions conferring ΦTE-resistance disrupted genes involved in flagellum biosynthesis (Table S1), suggesting that the host receptor for ΦTE was the flagellum. Restriction enzyme digestion analysis of the extracted ΦTE genome showed that it was terminally redundant and circularly permuted (Figure S1).

### ΦTE can evolve to escape ToxIN-mediated abortive infection

Serial dilutions of ΦTE were plated into top-lawns of Pba containing ToxIN plasmid pTA46 [11]. The Efficiency of Plating (EOP) of ΦTE was ∼1×10^−8^ compared with the ToxIN-frameshift (ToxIN-FS) negative controls. Phages were then isolated from six individual ΦTE plaques that formed on Pba (pTA46). These phages were plaque purified and passaged twice by growth on Pba without the ToxIN plasmid, and re-tested for sensitivity to ToxIN. The six mutant phage strains, denoted ΦTE-A to -F, plated on Pba (pTA46) with EOPs of 1.01, 1.07, 1.23, 0.90, 1.08 and 1.30, respectively, indicating that they had heritable mutations allowing them to ‘escape’ Abi by ToxIN. To identify the escape mutation, the genomes of ΦTE wild type (wt) and three escape ΦTE strains were sequenced.

### Genomic sequencing of wild-type and escape strains of ΦTE

Genomic DNAs were extracted from ΦTE, ΦTE-A, ΦTE-C and ΦTE-E and subjected to 454 sequencing. ΦTE had a dsDNA genome of 142,349 bp encoding 242 putative ORFs and two tRNAs (Figure 1C and Table S2). The coding regions of the phage were divided into four gene clusters, carried on both strands (Figure 1C).

Whilst 44% of ΦTE gene products had no detectable similarity with any protein in the NCBI database, a further 27% shared significant amino acid sequence identity with proteins encoded by the *Salmonella* phage PVP-SE1 [24] (Table S2). In many of these cases, the second most significant hit came from *Escherichia coli* phage rv5 (GenBank Accession no. DQ832317), which is the closest relative to PVP-SE1 [24]. PVP-SE1 has a similarly-sized genome to ΦTE, a similar morphology and is also terminally redundant and circularly permuted [24]. The tBLASTx results comparing ΦTE with PVP-SE1 and rv5 are shown (Figure 1C). Altogether, this suggests that ΦTE should be included within the “rv5-like virus” genus of the *Myoviridae* [24].

### ΦTE escape phages expand a repetitive antitoxin sequence

**Figure 2:**
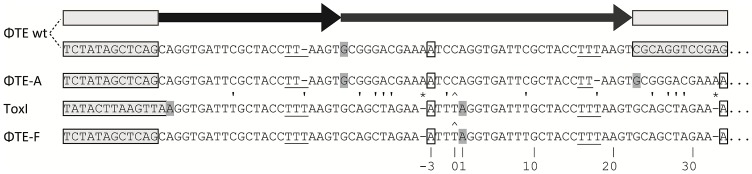
DNA alignment of ΦTE-phage escape loci and comparison with ToxI. Long grey boxes enclose invariant sequences bordering the pseudo-ToxI repeats. Grey shaded bases indicate the start of a DNA repeat. Single, boxed, bases mark the start of a ToxI antitoxic RNA and the predicted equivalent pseudo-ToxI RNAs in the ΦTE phages. Prime symbols denote single base-pair differences between sequences. Asterisks indicate the single base addition at the end of each pseudo-ToxI RNA repeat. A circumflex, ∧, indicates the single variable base in ToxI, where the first repeat shows a T in this position rather than a C for all other repeats. The numbering system identifies the 36 nucleotides within an antitoxic ToxI pseudoknot, numbered according to position relative to the DNA repeat, thereby beginning at base −3, −2, −1, 0 and through to base 32 [13]. Underlined bases denote the variable 2T or 3T sequences.

**Figure 3:**
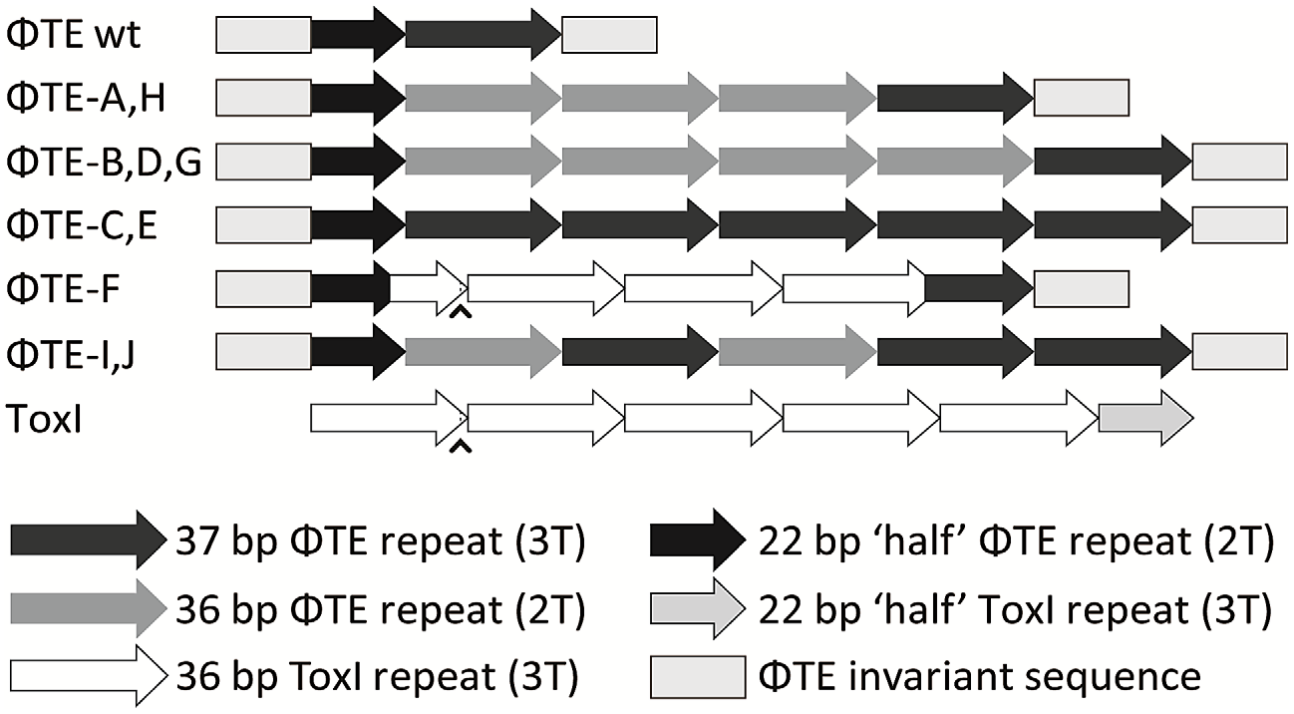
Schematic of the ΦTE-phage escape loci and ToxI. Each escape phage has expanded the number of DNA repeats, whilst ΦTE-F has recombined with *toxI*. The first ToxI repeat encoded by ΦTE-F matches the first ToxI repeat of pECA1039, as can be seen by the presence of the non-consensus T (∧) and dotted lines. Full sequences are in Table S3.

Escape phage ΦTE-A had a final genome size of 142,457 bp, whilst ΦTE-C and ΦTE-E were siblings with an identical genome of 142,497 bp. By comparing the finished sequences of ΦTE and the escape phages, a single difference was identified; the escape genomes had expanded in size within the intergenic locus between phiTE_202 and phiTE_203. This ‘escape locus’ between 106,714–106,772 bp of ΦTE wt (Figure 1C), contained a nucleotide sequence closely resembling that of the ToxI antitoxin from ToxIN (Figure 2). Within the escape phages, the number of DNA repeats had been expanded from 1.5 repeats to 4.5 (ΦTE-A) or 5.5 (ΦTE-C and -E; Figure 3, Table S3). The remaining escape phages were analysed by specifically sequencing amplicons generated from these escape loci. In phages ΦTE-B and ΦTE-D, the locus had also expanded to 5.5 repeats. The nature of each repeat varied in one position, in that a trinucleotide ‘TTT’ (denoted 3T) sometimes appeared in a repeat as a dinucleotide ‘TT’ (denoted 2T) (Figure 2 and Table S3). This difference separated ΦTE-B and -D from ΦTE-C and -E.

To ensure this escape response was not an isolated phenomenon, a second round of escape phage selection was performed. The escape phenotype of four new isolates, ΦTE-G to -J, was checked by plating the phages on Pba (pTA46); they plated with EOPs of 1.38, 1.67, 1.43 and 1.62, respectively. The escape loci of these four new escape phages were sequenced. Though isolated four months apart, we found that ΦTE-G had the same mutation as ΦTE-B and -D, whilst ΦTE-H had the same mutation as ΦTE-A. This suggests that these phages, though genetically identical, are not true siblings from a single escape event, and that an identical escape route can be taken in successive rounds of selection. This second batch also provided a new expansion, for phages ΦTE-I and -J (Figure 3 and Table S3).

One interesting outlier from the first isolation, ΦTE-F, appeared to have recombined with the ToxIN plasmid used during selection and incorporated ToxI into its genome, partially losing the ΦTE ‘pseudo-ToxI’ sequence in the process (Figure 2 and Figure 3). This recombination event generated mosaic repeats; the initial and final ‘repeats’ contained sequences from both pseudo-ToxI and ToxI itself (Figure 3 and Table S3).

### Functional analysis of the escape locus from ΦTE

**Figure 4:**
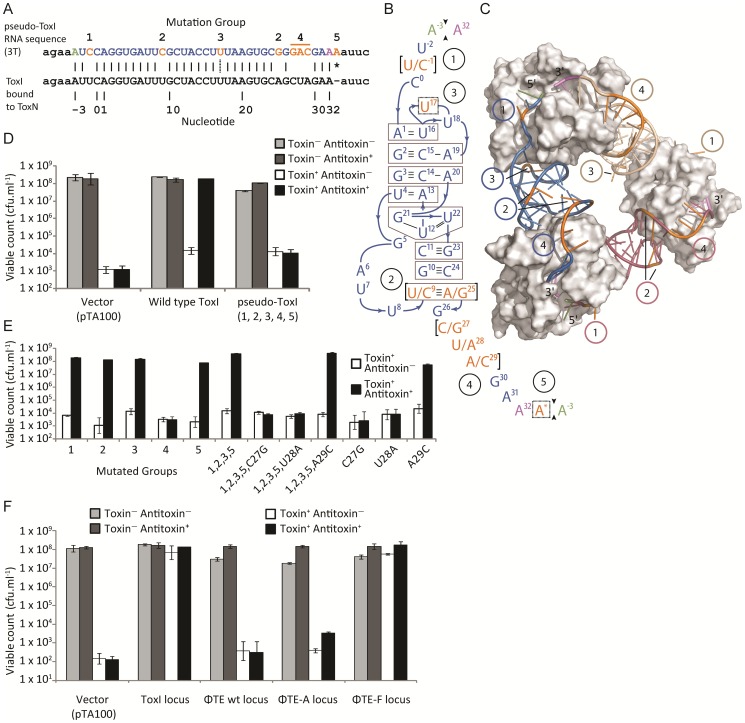
Analysis of pseudo-ToxI as a potential antitoxin. (A) Alignment of the pseudo-ToxI and ToxI RNA sequences. Pseudo-ToxI nucleotides are coloured to match (B) and (C), with the green and purple bases denoting the 5′ and 3′ ends of a single pseudoknot, respectively. Mutated nucleotides in pseudo-ToxI are coloured orange and numbered according to their grouping, whilst the asterisk indicates the additional 3′ nucleotide. The dotted line connecting the U in group 3 indicates the uracil that is deleted in the case of expanded repeats with 2T sequences rather than 3T. (B) Schematic of the ToxI pseudoknot. Each position containing a mutation in the pseudo-ToxI RNA has been bracketed, with the ToxI base separated from the pseudo-ToxI base by a ‘/’. The mutations have been grouped 1–5, according to position, and highlighted in orange, with the 5′ and 3′ termini in green and violet, respectively. Indels, such as U17 that is deleted in some pseudo-ToxI repeats, and the additional A* inserted in all, have been bordered by a dashed line. Base interactions are indicated by black lines, and duplex and triplex base-interactions are bordered in grey. (C) Detail of the ToxIN trimer with each pseudoknot shown either in blue, purple or beige. Each ToxN monomer is shown as a grey surface. The blue pseudoknot is oriented relative to (B). The positions of mutation groups are shown, with the group number encircled in the same colour as the corresponding pseudoknot. The additional nucleotide of group 5 is not visible as this was not in the original solved ToxIN structure. PDB: 2XDB. (D) Pseudo-ToxI cannot protect from ToxN in an over-expression assay. Protection assays were conducted as per Materials and Methods using strains of *E. coli* DH5α carrying both pTA49 (inducible ToxN) and a second inducible antitoxin vector as shown, including use of pTA100 as a vector-only control, “vector”. Error bars indicate the standard deviation of triplicate data. (E) Protection assays using mutants of ToxI carried out as in (D) with the antitoxin mutations in each construct numbered as per (B). (F) Protection assays carried out as in (D), testing the full escape loci of ΦTE wt, ΦTE-A and ΦTE-F with full ToxI as a positive control. Under these conditions, there was sufficient antitoxin present to inhibit induced ToxN even without specific induction of the ToxI and ΦTE-F constructs.

An alignment of the ToxI RNA sequence with the predicted pseudo-ToxI RNA showed that the majority of nucleotide positions were conserved (Figure 4A). When the mutated positions were mapped onto the structure of antitoxic ToxI RNA in complex with the ToxN protein [13], they formed five groups as defined by their proximity within the structure (Figure 4B and 4C). To ascertain whether the aligned pseudo-ToxI was in fact a functional antitoxin, a single repeat was cloned into an expression vector, pTA100 [11] and tested for the ability to inhibit ToxN over-expressed from a second, pBAD30-based [25] vector, within an *E. coli* model (Figure 4D). In this assay, pseudo-ToxI could not suppress ToxN toxicity (Figure 4D). Each mutation group was therefore examined in isolation to compare the contribution each made to rendering pseudo-ToxI inactive within this assay (Figure 4E). Groups 1, 2, 3 and 5 had no impact on the antitoxic activity of ToxI, alone or in concert, whilst group 4 was solely responsible for knocking-out ToxI function (Figure 4E). Group 4 comprised three contiguous nucleotides. The contribution of each individual group 4 nucleotide substitution was assessed. Mutations C27G and U28A were each independently sufficient to knock-out ToxI, either in the pseudo-ToxI (ie. with mutations 1, 2, 3 and 5) or ToxI backgrounds (Figure 4E). As pseudo-ToxI was not active as a single, aligned, repeat (Figure 4D), it was also decided to clone and test the whole escape loci from ΦTE wt, ΦTE-A and ΦTE-F (Figure 4F). Only the sequence from ΦTE-F (which had recombined with ToxI) was antitoxic (Figure 4F).

**Figure 5:**
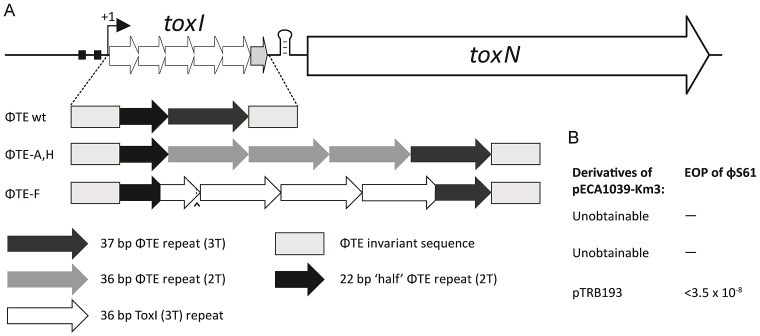
Only the recombinant ΦTE-F escape locus can replace ToxI in the native ToxIN locus. (A) Organisation of the ToxIN operon. Promoter elements are shown as black boxes. The transcriptional start site is indicated by the arrow with ‘+1’. The 5.5 repeats of *toxI* are followed by a stem-loop terminator structure, then *toxN*. It was possible to excise the full *toxI* sequence and then attempt to replace it with the escape locus sequences, including the invariant ends, from ΦTE wt, ΦTE-A and ΦTE-F, as shown in Figure 2. (B) The single plasmid that could be successfully generated, which included the insert from ΦTE-F, was tested for Abi activity against ΦS61 and seen to be highly active.

Finally, in attempts to generate a more native context, the escape loci from ΦTE wt, ΦTE-A and ΦTE-F were cloned to replace ToxI within the native *toxIN* operon (Figure 5A). This cloning required the use of a transposon-marked derivative of the original ToxIN plasmid, pECA1039-Km3 [11]. In this case, only recombinants with the insert from ΦTE-F could be obtained, presumably due to toxic effects preventing cloning of ΦTE wt and ΦTE-A. The ΦTE-F plasmid retained Abi activity (Figure 5B), as tested with ΦS61 [12], which was used in order to prevent any possible interaction of an infecting ΦTE with the regions of the ΦTE genome cloned onto the test plasmid construct.

### Expression of pseudo-ToxI antitoxin can inhibit abortive infection

**Figure 6:**
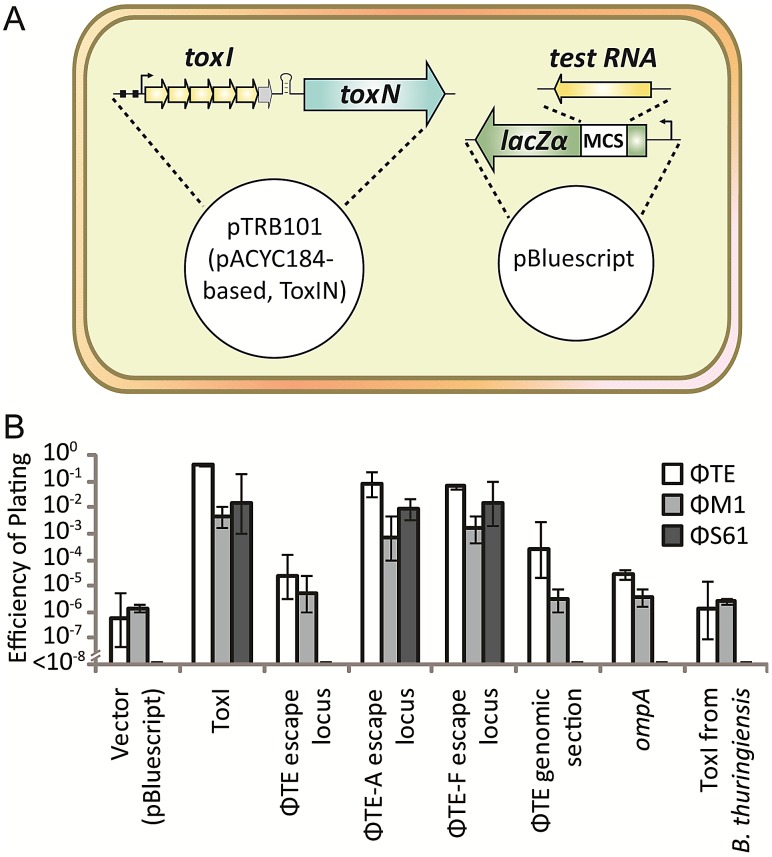
An excess of pseudo-ToxI inhibits abortive infection. (A) Strains of Pba ToxIN (pTRB101) were tested for their ability to abort infection in the presence of a second, pBluescript II SK- based, antitoxic plasmid. Putatively antitoxic “test RNA” sequences were cloned under the control of the constitutive *lacZα* promoter, to allow for constant, high-level, expression. (B) EOPs of ΦTE, ΦM1 and ΦS61 on double strains of Pba, as per key, using Pba ToxIN-FS (pTRB102, pBluescript II SK-) as the control strain. Inserts in the second, antitoxic, plasmids are indicated by the horizontal axis labels. Plasmid pBluescript II SK- was used as the no insert, “vector”, control. “ΦTE escape locus” includes the escape locus from wild type ΦTE, whilst the “ΦTE genomic section” is a 269 bp region of the ΦTE genome, taken several kb from the escape locus as a negative control. Error bars indicate the standard deviation of triplicate (minimum) data.

As the protection assays above (Figure 4D and 4E) relied on over-expression of the toxic and antitoxic components, it was considered that they may act as poor approximations to the relative stoichiometries of ToxN, ToxI and pseudo-ToxI during ΦTE infections. An assay was therefore designed to focus on whether Abi could be perturbed by excess antitoxin (Figure 6A). An excess was generated by first cloning both control and ΦTE-derived antitoxin sequences into the multiple cloning site of the high copy-number plasmid pBluescript II SK- (Fermentas), oriented so that they would be transcribed from the *lacZα* promoter. This constitutive promoter would ensure an over-abundance of our test RNA. These constructs were used in attempts to alter the Abi phenotype of an independent, pACYC184-based [26] ToxIN plasmid (pTRB101) within Pba.

In the presence of the positive control ToxI plasmid, Abi against ΦTE was greatly suppressed, compared to the vector control (Figure 6B). Escape locus sequences from ΦTE-A and -F were also able to suppress Abi to similar levels as ToxI, whilst the same locus from ΦTE wt did not have an effect (Figure 6B). To confirm that this result was dependent on the specific sequence cloned into pBluescript, further negative controls were tested; a section of the ΦTE genome upstream of the escape locus (269 bp, from 99,267 to 99,536 bp), the *E. coli* coding sequence for *ompA* (a known substrate of ToxN) [13] and a non-cognate antitoxic ToxI from the ToxIN system of *Bacillus thuringiensis*. None of these three negative controls made any significant impact on the Abi phenotype of ToxIN against ΦTE (Figure 6B). To confirm that this was not a phage-specific effect, the experiments were repeated with phages ΦM1 and ΦS61 [12]. The same trend was observed, with protection from Abi only observed with plasmids containing either the escape loci of ΦTE-A and -F, or cognate ToxI (Figure 6B). The effect of ToxIN upon ΦS61 was so pronounced that, when no protection was provided from the test construct, no phage plaques could be observed. This produced an EOP of <1×10^−8^ (Figure 6B) and provided a stark indication of which constructs acted as antitoxins. Collectively, these data show that Abi can be suppressed by an expanded pseudo-ToxI sequence, or cognate ToxI, within the infected cell.

### ΦTE expresses antitoxin mimics during infection

**Figure 7:**
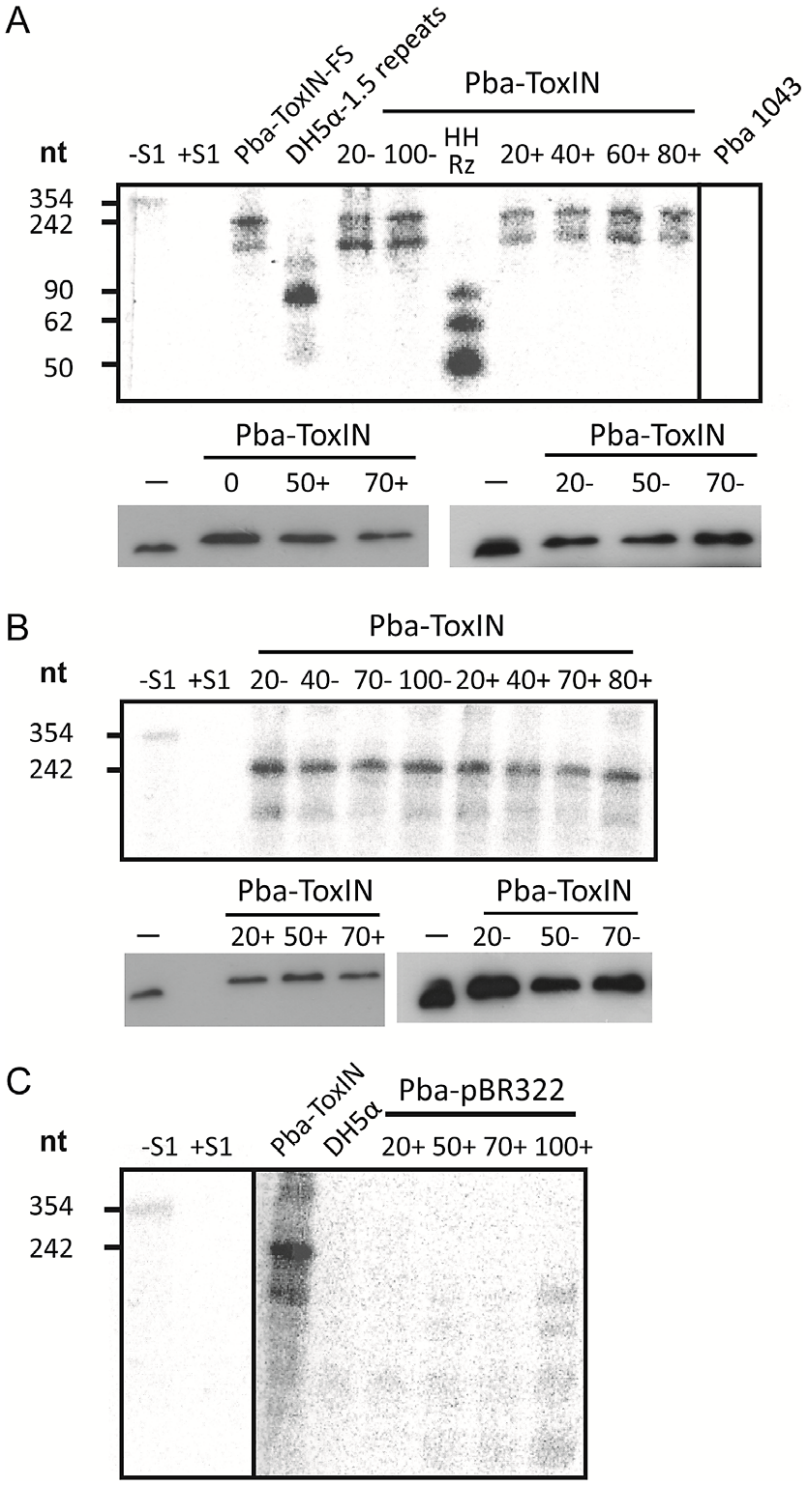
ΦTE-F expresses ToxI RNA during infection. (A) Upper panel; an S1-nuclease protection assay was used to detect ToxI levels from a ToxIN plasmid during ΦTE wt infection, using an antisense probe against the full 5.5 repeat ToxI sequence [46]. The antisense ToxI-probe was first hybridised to 10 µg of total RNA prepared from Pba ToxIN (pMJ4) at different times after ΦTE infection, and then followed by S1-nuclease treatment. Numbers (+) indicate the time (min) after infection or (−) without the addition of phage. Pba ToxIN-FS (pTA47) and Pba serve as positive and negative controls, respectively. A non-hybridized S1-digested probe (+S1) serves as a further negative control. DH5α 1.5 repeats (pTA96), a non-S1 digested probe (−S1) and an *in vitro* transcribed Hammerhead ribozyme (HHRz), which cleaves itself during transcription, serve as size markers. HHRz was prepared as described previously [45]. Lower panel; Western blot targeting C-terminal FLAG tagged ToxN contained within total protein harvested from Pba ToxIN (pMJ4) at different time points, with (+, left) and without (−, right) phage infection. Time 0 indicates a sample taken immediately after infection. Total protein from Pba ToxIN (pMJ4) (−) serves as positive control. (B) Infection with escape phage ΦTE-F. Levels of ToxI were determined by S1-assay (upper) as described in (A) with and without infection. ToxN levels were estimated by Western blotting (lower) as described in (A). (C) Expression of the ΦTE-F ToxI locus. An S1-nuclease assay targeting ToxI was performed on total RNA of Pba (pBR322) at different times during ΦTE-F infection. Pba ToxIN (pMJ4) and DH5α serve as positive and negative controls, respectively.

Having ascertained that expression of pseudo-ToxI during infection could alter the Abi outcome, we studied the levels of antitoxic RNA and ToxN protein during ΦTE infections of Pba containing FLAG-tagged ToxIN plasmid pMJ4 [12]. When infecting Pba with ΦTE wt, there were no detectable differences in ToxI or ToxN levels between the infected and uninfected controls, as measured by S1 nuclease assays and Western blotting of the ToxN-FLAG protein, respectively (Figure 7A). To see if an ‘escape’ ΦTE phage caused an alteration in the ToxI:ToxN ratio, the same experiment was performed with phage ΦTE-F. No detectable differences were observed following infection relative to the uninfected controls (Figure 7B).

Using the same probe, it was then possible to examine whether ToxI was expressed from the recombinant escape phage ΦTE-F, during infection of Pba carrying a vector-only pBR322 (NEB) control rather than a ToxIN plasmid. Again, this was monitored by taking regular total cellular RNA samples during an infection cycle and detecting the escape transcripts of phage ΦTE-F by S1 nuclease assay (Figure 7C). These assays showed specific detectable transcripts from the ΦTE-F escape locus that increased during phage infection. The transcripts from the ΦTE-F escape locus were shorter, and expressed at a lower level, than observed for the Pba-ToxIN (pMJ4) control (Figure 7C). The shorter length was consistent with the differences between the ΦTE-F escape locus and ToxI.

### Generalised transduction of ToxIN by ΦTE

ΦTE was tested for the ability to transduce selectable markers. ΦTE was able to transduce chromosomal markers from Pba strains SCC34 and SCC14 [27] along with plasmids pKD46 [28] and pBluescript II KS^+^ (Table 1). ΦTE wt and escape phages ΦTE-A and -F were then used in attempts to transduce plasmids pTRB101 (ToxIN) and pTRB102 (ToxIN-FS). Phages ΦTE-A and ΦTE-F plated on pTRB101 with an EOP of ∼1, whereas ΦTE wt had an EOP of 1×10^−7^. It was therefore necessary to plate ∼10^7^ more ΦTE wt phages to obtain a pTRB101 transducing lysate, than with the two isolated escape phage strains. Once generated, however, the ΦTE wt pTRB101 lysate then transduced approximately as efficiently as the ΦTE-A and -F lysates (Table 1). The resulting pTRB101 transductant strains were then confirmed as having gained Abi activity. Finally, ΦTE-F was used to transduce a marked derivative of the native ToxIN plasmid, pECA1039, proving that phage-mediated horizontal transmission of the naturally-occurring phage-resistance replicon was also possible (Table 1).

**Table 1:** ΦTE-mediated transduction of different markers into Pba 1043.

Phage	Donor strain	Marker^a^	Mean relative frequency of transduction
ΦTE	SCC34	Km	1.33×10^−9^
ΦTE	SCC14	*virS::Km*	1.94×10^−9^
ΦTE	Pba 1043 pKD46	Ap	1.40×10^−8^
ΦTE	Pba 1043 pBluescriptII KS+	Ap	1.16×10^−9^
ΦTE	Pba 1043 pTRB101 (pACYC184-ToxIN)	Cm	1.86×10^−7^
ΦTE	Pba 1043 pTRB102 (pACYC184-ToxI, ToxN-FS)	Cm	4.40×10^−9^
ΦTE-A	Pba 1043 pTRB101 (pACYC184-ToxIN)	Cm	1.43×10^−8^
ΦTE-A	Pba 1043 pTRB102 (pACYC184-ToxI, ToxN-FS)	Cm	1.47×10^−8^
ΦTE-F	Pba 1043 pTRB101 (pACYC184-ToxIN)	Cm	2.07×10^−8^
ΦTE-F	Pba 1043 pTRB102 (pACYC184-ToxI, ToxN-FS)	Cm	1.10×10^−8^
ΦTE-F	Pba 1043 pECA1039-Km12 (pECA1039 EZ:TN derivative, ToxIN^+^)	Km	1.90×10^−10^
ΦTE-F	Pba 1043 pECA1039-Km23 (pECA1039 EZ:TN derivative, ΔToxN)	Km	4.01×10^−9^

a Ap, ampicillin; Cm, chloramphenicol; Km, kanamycin.

## Discussion

We isolated and characterised phage ΦTE, a flagellum-dependent generalised transducing *Myoviridae* member of the “rv5-like virus” genus (Figure 1 and Figure S1). The genome of ΦTE did not contain any evidence of a lysogeny module or remnant integrases, confirming ΦTE to be a lytic phage. ΦTE was shown to undergo Abi by the Type III TA system, ToxIN. Abi resulted in the selection of ToxIN-insensitive mutant ΦTE ‘escape’ phages. Genome sequencing identified a ΦTE ‘escape locus’ containing a repetitive DNA sequence, which was similar to the repetitive 36 nucleotide sequence of each unit from the ToxI antitoxic non-coding RNA (Figure 2 and Figure 3; Table S3). ΦTE wt contained 1.5 repeats of this ‘pseudo-ToxI’ sequence, whilst the escape phages had expanded this number (Figure 3). In one case, ΦTE-F, a recombination event had occurred between the phage genome and the plasmid carrying *toxIN* (Figure 2 and Figure 3). Alignment with, and consequent mutagenesis of, ToxI, identified those mutations of pseudo-ToxI which impaired antitoxic activity within over-expression assays (Figure 4). Next, we developed a novel Abi inhibition assay, in which a cell is pre-loaded with potentially antitoxic RNAs and then tested to see whether this abundance of RNA would be sufficient to inhibit the ToxN protein and thereby Abi (Figure 6A). This assay showed that pseudo-ToxI adequately mimicked ToxI activity and suppressed ToxN anti-phage activity *in vivo* (Figure 6B). Biochemical study of ToxI RNA levels during ΦTE-F infection indicated that pseudo-ToxI would indeed be expressed during the replication cycle of ΦTE phages (Figure 7). ΦTE was also shown to be able to transduce the native ToxIN plasmid (Table 1).

By comparing the nucleotide sequence from a single repeat of pseudo-ToxI with that of ToxI (Figure 4A) and considering how the mutations may alter the shape of the resulting RNA pseudoknot (Figure 4B and 4C), it became clear how pseudo-ToxI may act to inhibit ToxN. The Group 1 mutation is U-1C (Figure 4B and 4C). This change is in the initial ssRNA tail, with the base clearly extending away from the complex (Figure 4C). The next alteration (denoted by, ∧, in Figure 2 and Figure 3) can be discounted as it only appears as a mutation against the first ToxI repeat; this has a non-consensus T rather than the consensus C, which would otherwise match pseudo-ToxI. Group 2 can be considered a compensatory set of mutations, as U9C is matched by A25G; together, they form the end duplex interaction of the pseudoknot central stack [13]. Variability in each pseudo-ToxI arises from whether the group 3 mutation is present, deleting the sequence ‘UUU’ to ‘UU’ at U16-U18. This region forms the second loop of the ToxI pseudoknot and is not vital for interaction with ToxN, so is likely to allow for some flexibility in length. Jumping ahead to group 5, this is an additional A at the 3′ end of each pseudo-ToxI, which conserves the sequence around the defined cleavage site. Mutations in groups 1, 2, 3 and 5 were all shown not to effect pseudo-ToxI antitoxicity (Figure 4E). This highlights the innate plasticity in pseudoknot formation and the interaction of a Type III antitoxin with its cognate toxic protein. There are limits to this plasticity, however, as group 4 contains knock-out mutations; positions 27–29 are CUA in ToxI, but GAC in pseudo-ToxI. Mutation A29C has no effect, but both C27G and U28A are individually sufficient to knock-out ToxI antitoxicity in over-expression assays (Figure 4E). This can be readily explained when examining the ToxIN structure, as both C27 and U28 are involved in extensive hydrogen-bond networks at the previously defined major interface between ToxI and ToxN [13].

From these analyses it is clear why it is necessary to expand the ΦTE escape locus in order to obtain an escape phenotype. Though the ΦTE wt locus has 1.5 DNA repeats, it does not encode one full pseudo-ToxI RNA (Figure 2). The phasing of the repetitive DNA and active RNA sequences differ, so that within the ΦTE wt locus, it is not possible to generate a pseudo-ToxI RNA that could readily form a pseudoknot. Only by expanding this locus can a phage then express active pseudo-ToxI RNAs that can fold properly to mimic cognate ToxI and suppress ToxN. We suggest that these expansions of pseudo-ToxI may have arisen through strand slippage during replication. When we previously cloned these regions, we often observed a range of PCR products containing 2.5 to 5.5 copies of the repeat sequences [11]. Strand slippage would help to generate the observed diversity in the number of repeats and mosaic nature of each expanded escape locus. This could also reflect the generation of ToxI in nature, and may help to explain the diversity in the number of repeats shown within the family of ToxI sequences [3]. This expansion to either 4.5 or 5.5 repeats also highlights how the levels of expression must be exquisitely tuned, in order to inhibit Abi within the native infection setting.

This observed mode of escape from Abi differs from either the specific point mutations observed in the *sak* and *sav* genes, (which allow lactococcal phages to escape AbiK and AbiV, respectively [17], [18]), or the extremely large-scale genomic re-arrangements observed in phages escaping AbiK and AbiT [29]. Here, ΦTE carries an inactive antitoxin which can be selectively upregulated through expansions in genomic DNA content. In the case of ΦTE-F, the phage actively recombined with the ToxIN Abi system to hijack the antitoxic component and protect itself from the host defences. A similar case was previously observed when a virulent phage transferred a host methylase gene into its genome, providing protection from the host restriction-modification system [30]. Within ΦTE-F, however, it is an RNA that provides the advantage. Large non-coding RNAs have been identified within prophages from a restricted number of bacterial species, and are thought to be involved in host lysis and virion production [31]. Selected temperate phages use non-coding RNAs to mediate superinfection immunity [32]. To the best of our knowledge this is the first example, however, where an infecting phage evolves a non-coding RNA to suppress host defensive mechanisms and ensure phage replication.

As the observed transduction efficiencies for ΦTE, though low, are similar to values for transducing phages of *Citrobacter rodentium* and *Pseudomonas aeruginosa* [33], [34], ΦTE would make a suitable tool for functional genomics. In the environment, the apparently low efficiency of transduction will be counter-acted by the abundant microbiological populations and high frequency of infection. This identification of ΦTE as a transducing phage also provided us with the opportunity to investigate transfer of ToxIN. Whilst conjugation has been used to transfer both chromosomally encoded AbiV [35] and other plasmid-based Abi systems [36], pECA1039 does not contain any obvious components for conjugal transfer [11]. Instead, we were able to show transduction of ToxIN plasmids by ΦTE phages, making this a key example of phage-mediated transfer of phage-resistance mechanisms. Given the mode of action of ToxIN, this particular case may demonstrate a *de facto* example of “infectious altruism”. This finding may also help to explain the widespread horizontal transfer of both chromosomal and plasmid-borne Type III TA systems observed in a recent bioinformatic study [3].

We can consider this three-way, phage-host-plasmid, interaction, from the point of view that ToxIN, as a toxin-antitoxin system, is an addictive and selfish element [8]. Selection of an escape phage would indeed have the negative effect of allowing more productive infection within host cells containing ToxIN, but it may also lead to increased dissemination of the ToxIN plasmid. Considering the selfish phage, this would then allow ΦTE escape phages to select against other, ToxIN-sensitive, phages competing for the same bacterial host. The interactivity between these three elements shapes the evolution of further methods of defence and counter-attack. It will be interesting to identify how other phages escape Abi by ToxIN and indeed whether these escape mechanisms are as widespread as the resistance mechanisms themselves.

## Materials and Methods

### Bacterial strains, bacteriophages, and growth conditions

Bacterial strains and bacteriophages are listed in Table S4. All experiments were performed with *E. coli* strain DH5α (Gibco/BRL) or *Pectobacterium atrosepticum* SCRI1043 [19] and derivatives thereof. Bacteriophage ΦTE was isolated from sewage effluent collected from the outlet at Milton sewage treatment plant, near Cambridge, UK. *E. coli* strains were grown at 37°C and Pba was grown either at 25°C on plates or at 25, 28, or 30°C as required for liquid culture, in Luria broth (LB) at 250 rpm or on LB-agar (LBA). LBA contained 1.5% w v^−1^ or 0.35% w v^−1^ agar, to make LBA plates or top-LBA, respectively. Growth (OD_600_) was measured using a spectrophotometer set to 600 nm. When required, media was supplemented with ampicillin (Ap) at 100 µg ml^−1^, chloramphenicol (Cm) at 50 µg ml^−1^, kanamycin (Km) at 50 µg ml^−1^ and spectinomycin (Sp) at 50 µg ml^−1^. Bacteriophages were isolated as described [23] though using SCC34 as the host strain. Phage lysates were made as described [37]. Phages were stored at 4°C in phage buffer; 10 mM Tris-HCl pH 7.4, 10 mM MgSO_4_, 0.01% w v^−1^ gelatin. Efficiency of Plating (EOP) was calculated after overnight incubation of phage lysate serial dilutions in a top-LBA lawn of each bacterial host, using (pfu on test strain/pfu on control strain).

### Electron microscopy

Electron microscopy was performed at the Multi-Imaging Centre, University of Cambridge, using a Tecnai G2 series transmission electron microscope. Samples were prepared by first placing charge-discharged copper grids onto 50 µl drops of high titre (≥1×10^10^ pfu ml^−1^) lysates of ΦTE for 1 min. Each grid was then washed briefly with three drops of distilled water, followed by staining with 1% phosphotungstic acid for 1 min. The accelerating voltage was 120.0 kV and the direct magnification was 25,000×.

### ΦTE genomic sequencing

Bacteriophage DNA was extracted with phenol/chloroform, using phase-lock gel tubes (Eppendorf) and following the manufacturer's instructions as for bacteriophage λ. The extracted DNA was subjected to pyrosequencing on a Roche 454 Genome Sequencer FLX at the DNA sequencing facility, Department of Biochemistry, University of Cambridge. Contigs were assembled using Newbler (Roche).

ORFs were identified in the ΦTE genome using Glimmer and LongORFS [38]. Homologues of predicted proteins were identified using BLASTp [39]. ΦTE tRNAs were identified using tRNAScan-SE [40]. RBSfinder [41] was used to identify ribosome binding sites (Table S2). The ΦTE genome was viewed and annotated using Artemis [42] and the GenBank submission file was generated using Sequin (NCBI). Figure 1C was generated by using Adobe Illustrator to adapt an output from the CGView Server [43].

### Plasmid construction

Molecular biology techniques were performed as described previously [44]. All primers were obtained from Sigma-Genosys and Invitrogen and are listed in Table S5. All plasmids constructed and/or used in this study are listed in Table S6, along with the primers used for their construction. All recombinant plasmid sequences were verified by DNA sequencing.

### Protection assays

When required, media was supplemented with Ap, Sp, D-glucose (glu) at 0.2% w v^−1^, L-arabinose (L-ara) at 0.1% w v^−1^ and isopropyl-β-D-thiogalactopyranoside (IPTG) at 1 mM. Double-plasmid carrying DH5α strains were grown as 10 ml overnight cultures, then used to inoculate 25 ml of LB, Ap and glu in 250 ml flasks, and grown at 37°C and 250 rpm, from a starting OD_600_ of ∼0.04, until exponential phase (∼1×10^8^ colony forming units (cfu) ml^−1^). Samples were removed, washed with phosphate buffered saline (PBS) serially diluted and plated for viable counts at 37°C on LBA, Ap, Sp plates containing either i) glu, so neither toxin or antitoxin were expressed; ii) glu and IPTG, to express the antitoxin; iii) L-ara, to express the toxin; or iv) L-ara and IPTG to express both the toxin and antitoxin.

### Measuring ToxI and ToxN levels during phage infection

Two cultures of 180 ml of LB containing Ap were inoculated with 2 ml of overnight cultures of Pba (pBR322) or Pba (pMJ4), respectively. Cultures were grown at 25°C and 180 rpm to an OD_600_ of 1 and each split into 2×80 ml; one of which was infected with phage to a multiplicity of infection (MOI) of 1 while the other served as a negative control without infection. Cultures were left 10 min without shaking for phage adsorption, then shaken at 25°C and 180 rpm. Samples for OD_600_ measurement, RNA preparation and protein analysis were taken regularly during infection. Total RNA was isolated using the TRIZOL method and subsequently DNase treated. Cell pellets for Western blot analysis were resuspended in 1× PBS according to OD_600_ measurement.

### Western blotting

One ml cell samples were taken, pelleted and resuspended in 1× PBS according to OD_600_. The protein was quantified using Nanodrop (ThermoScientific) and equal amounts of protein (150 µg) were resolved by 12% PAGE. Proteins were transferred to a PVDF-membrane and blocked for 1 hour in 1× PBS containing 5% milk powder. Immunodetection of FLAG-tagged ToxN was performed overnight at 4°C in 1× PBS using anti-FLAG M2 antibody (Sigma). Goat anti-mouse IgG-HRP (Santa Cruz) was used as secondary antibody. Bands were visualised on X-Ray film using the SuperSignal West Pico Chemiluminescent Substrate Kit (Pierce).

### Construction of size markers for S1-nuclease assays

Plasmid pTA96 was generated to act as a size marker in the S1-nuclease assays, using overlap extension PCR. Briefly, the 5′ flanking fragment encoding the *toxIN* promoter was amplified using pTA47 as template with PF221 and PF162. The 3′ flanking fragment containing 1.5 *toxI* repeats and *toxN*-FS was amplified using PF168 and PF222 and pTA47 as template. The overlap PCR used PF221 and PF222 and the 5′ and 3′ flanking sequences as templates, the product was digested with EcoRI and HindIII and cloned into EcoRI/HindIII digested pBR322. A second size marker, HHRz, was prepared as described [45].

### S1-nuclease protection assays

An antisense probe covering the complete ToxI sequence was made by amplification of the ToxI locus from plasmid pTA110, using primers PF217 and PF218, and subsequent *in vitro* transcription and gel extraction of the probe as described [46] generating a uniformly ^32^P-UTP labeled antisense transcript. Ten g of DNase-treated total RNA was hybridised to the antisense probe overnight at 68°C in a total volume of 30 µl containing 38% or 23% formamide for the ΦTE and ΦTE-F total RNA, respectively, 40 mM PIPES/KOH (pH 6.4), 1 mM EDTA and 400 mM NaCl. Reactions were treated with S1-nuclease (Invitrogen) (1 U µl^−1^) for 1.5 hr at 37°C in a total volume of 300 µl of 1× S1-nuclease buffer, to degrade any single-stranded nucleic acids. Double-stranded hybridisation products were precipitated, resuspended and resolved by 10% PAGE. Bands were visualised by phosphorimaging (BioRad Personal FX phosphorimager).

### Generalised transduction using ΦTE

High titre (≥1×10^10^ pfu ml^−1^) lysates of ΦTE were prepared on the bacterial donor strain. An appropriate volume of the transducing lysate (either 10 or 100 µl) was added to 5 ml overnight cultures of the recipient strain. This was incubated on a roller wheel at 30°C for 45–60 min. The cells were then pelleted and the supernatant was removed. The remaining pellet was resuspended in 250 µl of LB and 100 µl of this suspension was plated onto LBA containing the appropriate selection. Controls for contamination and spontaneous antibiotic resistance were performed by also plating samples of the phage lysates and samples of the recipient strains inoculated with phage buffer, respectively. Any transductants obtained were streaked out twice prior to use, to reduce any bacteriophage carry-over. Transductants receiving ToxIN plasmids pTRB101 or pECA1039-Km12 were also confirmed as having Abi activity, using phage ΦTE wt for pTRB101 and both ΦTE wt and ΦM1 for pECA1039-Km12.

### Additional methods

Further detailed methods for characterisation of the ΦTE genome are available in Text S1.

## Supporting Information

Figure S1 Characterisation of the ΦTE genome (A–C) Restriction digestion of ΦAT1, ΦM1 and ΦTE genomic DNAs. (A) Identification of *cos* sites. Lanes 1, 4, 7, 10: ΦAT1; lanes 2, 5, 8, 11: ΦTE; lanes 3, 6, 9, 12: ΦM1. Lanes 7–12 are the same as lanes 1–6 except digestion reactions were heated to 65°C for 20 min before loading to attempt to identify *cos* sites; none were evident. (B) Phage genomic DNA was treated with a Bal-31 exonuclease time course before restriction digestion to determine if the genome was circularly permuted. Numbers indicate the length of Bal-31 treatment (min). Specific bands were observed to be preferentially degraded by Bal-31 treatment of ΦAT1 and ΦM1, suggesting these two phages are not circularly permuted (red boxes). The sub-molar fragment in the ΦTE digest could represent the *pac* fragment, and is indicated with a red arrow. (C) ΦTE is circularly permuted. ΦTE digest, as in (B), but with longer incubation, showing that all restriction fragments are lost following Bal-31 treatment, proving that ΦTE has a circularly permuted genome. ‘M’ indicates DNA size markers in Kb (1 Kb ladder, Invitrogen).
(TIF)

Table S1 Transposon insertion sites within ΦTE-resistant strains.
(DOCX)

Table S2 Details of ORFs, tRNAs and ncRNA within ΦTE wild-type genome.
(DOCX)

Table S3 Full sequences and details of ΦTE escape loci.
(DOCX)

Table S4 Bacterial strains and bacteriophages used in this study.
(DOCX)

Table S5 Primers used in this study.
(DOCX)

Table S6 Plasmids used in this study.
(DOCX)

Text S1 Contains further Results, Materials and Methods, and References.
(DOCX)

## References

[1] Fozo EM, Makarova KS, Shabalina SA, Yutin N, Koonin EV, et al (2010) Abundance of type I toxin-antitoxin systems in bacteria: searches for new candidates and discovery of novel families. Nucl Acids Res 38: 3743–3759. 20156992 10.1093/nar/gkq054 PMC2887945

[2] Leplae R, Geeraerts D, Hallez R, Guglielmini J, Dreze P, et al (2011) Diversity of bacterial type II toxin-antitoxin systems: a comprehensive search and functional analysis of novel families. Nucl Acids Res 39: 5513–5525. 21422074 10.1093/nar/gkr131 PMC3141249

[3] Blower TR, Short FL, Rao F, Mizuguchi K, Pei XY, et al (2012) Identification and classification of bacterial Type III toxin-antitoxin systems encoded in chromosomal and plasmid genomes. Nucl Acids Res doi:10.1093/nar/gks231 10.1093/nar/gks231 PMC3401426 22434880

[4] Blower TR, Salmond GP, Luisi BF (2011) Balancing at survival's edge: the structure and adaptive benefits of prokaryotic toxin-antitoxin partners. Curr Opin Struc Biol 21: 109–118. 10.1016/j.sbi.2010.10.009 21315267

[5] Ogura T, Hiraga S (1983) Mini-F plasmid genes that couple host cell division to plasmid proliferation. Proc Natl Acad Sci U S A 80: 4784–4788. 6308648 10.1073/pnas.80.15.4784 PMC384129

[6] Gerdes K, Christensen SK, Lobner-Olesen A (2005) Prokaryotic toxin-antitoxin stress response loci. Nat Rev Microbiol 3: 371–382. 15864262 10.1038/nrmicro1147

[7] Maisonneuve E, Shakespeare LJ, Jorgensen MG, Gerdes K (2011) Bacterial persistence by RNA endonucleases. Proc Natl Acad Sci U S A 108: 13206–13211. 21788497 10.1073/pnas.1100186108 PMC3156201

[8] Magnuson RD (2007) Hypothetical functions of toxin-antitoxin systems. J Bacteriol 189: 6089–6092. 17616596 10.1128/JB.00958-07 PMC1951896

[9] Pecota DC, Wood TK (1996) Exclusion of T4 phage by the *hok/sok* killer locus from plasmid R1. J Bacteriol 178: 2044–2050. 8606182 10.1128/jb.178.7.2044-2050.1996 PMC177903

[10] Hazan R, Engelberg-Kulka H (2004) *Escherichia coli mazEF*-mediated cell death as a defense mechanism that inhibits the spread of phage P1. Mol Genet Genomics 272: 227–234. 15316771 10.1007/s00438-004-1048-y

[11] Fineran PC, Blower TR, Foulds IJ, Humphreys DP, Lilley KS, et al (2009) The phage abortive infection system, ToxIN, functions as a protein-RNA toxin-antitoxin pair. Proc Natl Acad Sci U S A 106: 894–899. 19124776 10.1073/pnas.0808832106 PMC2630095

[12] Blower TR, Fineran PC, Johnson MJ, Toth IK, Humphreys DP, et al (2009) Mutagenesis and functional characterisation of the RNA and protein components of the toxIN abortive infection and toxin-antitoxin locus of *Erwinia* . J Bacteriol 191: 6029–6039. 19633081 10.1128/JB.00720-09 PMC2747886

[13] Blower TR, Pei XY, Short FL, Fineran PC, Humphreys DP, et al (2011) A processed non-coding RNA regulates an altruistic bacterial antiviral system. Nat Struc Mol Biol 18: 185–190. 10.1038/nsmb.1981 PMC4612426 21240270

[14] Emond E, Holler BJ, Boucher I, Vandenbergh PA, Vedamuthu ER, et al (1998) AbiQ, an abortive infection mechanism from *Lactococcus lactis* . Appl Environ Microbiol 64: 4748–4756. 9835558 10.1128/aem.64.12.4748-4756.1998 PMC90918

[15] Labrie SJ, Samson JE, Moineau S (2010) Bacteriophage resistance mechanisms. Nat Rev Microbiol 8: 317–327. 20348932 10.1038/nrmicro2315

[16] Chopin MC, Chopin A, Bidnenko E (2005) Phage abortive infection in lactococci: variations on a theme. Curr Opin Microbiol 8: 473–479. 15979388 10.1016/j.mib.2005.06.006

[17] Bouchard JD, Moineau S (2004) Lactococcal phage genes involved in sensitivity to AbiK and their relation to single-strand annealing proteins. J Bacteriol 186: 3649–3652. 15150253 10.1128/JB.186.11.3649-3652.2004 PMC415755

[18] Haaber J, Rousseau GM, Hammer K, Moineau S (2009) Identification and characterization of the phage gene *sav*, involved in sensitivity to the lactococcal abortive infection mechanism AbiV. Appl Environ Microbiol 75: 2484–2494. 19270128 10.1128/AEM.02093-08 PMC2675237

[19] Bell KS, Sebaihia M, Pritchard L, Holden MT, Hyman LJ, et al (2004) Genome sequence of the enterobacterial phytopathogen *Erwinia carotovora* subsp. *atroseptica* and characterization of virulence factors. Proc Natl Acad Sci U S A 101: 11105–11110. 15263089 10.1073/pnas.0402424101 PMC503747

[20] Toth IK, Mulholland V, Cooper V, Bentley S, Shih Y-L, et al (1997) Generalized transduction in the potato blackleg pathogen *Erwinia carotovora* subsp. *atroseptica* by bacteriophage ΦM1. Microbiology 143: 2433–2438. 10.1099/00221287-143-7-2433 33657724

[21] Evans TJ (2009) Investigation of bacteriophages and their use in the analysis of enterobacterial virulence. Ph.D Thesis, Department of Biochemistry, University of Cambridge, UK.

[22] Ackermann HW, DuBow MS, Gershman M, Karska-Wysocki B, Kasatiya SS, et al (1997) Taxonomic changes in tailed phages of enterobacteria. Arch Virol 142: 1381–1390. 9267450

[23] Evans TJ, Trauner A, Komitopoulou E, Salmond GP (2010) Exploitation of a new flagellatropic phage of *Erwinia* for positive selection of bacterial mutants attenuated in plant virulence: towards phage therapy. J Appl Microbiol 108: 676–685. 19674185 10.1111/j.1365-2672.2009.04462.x

[24] Santos SB, Kropinski AM, Ceyssens PJ, Ackermann HW, Villegas A, et al (2011) Genomic and Proteomic Characterization of the Broad-Host-Range *Salmonella* Phage PVP-SE1: Creation of a New Phage Genus. J Virol 85: 11265–11273. 21865376 10.1128/JVI.01769-10 PMC3194984

[25] Guzman LM, Belin D, Carson MJ, Beckwith J (1995) Tight regulation, modulation, and high-level expression by vectors containing the arabinose P_BAD_ promoter. J Bacteriol 177: 4121–4130. 7608087 10.1128/jb.177.14.4121-4130.1995 PMC177145

[26] Chang AC, Cohen SN (1978) Construction and characterization of amplifiable multicopy DNA cloning vehicles derived from the P15A cryptic miniplasmid. J Bacteriol 134: 1141–1156. 149110 10.1128/jb.134.3.1141-1156.1978 PMC222365

[27] Liu H, Coulthurst SJ, Pritchard L, Hedley PE, Ravensdale M, et al (2008) Quorum sensing coordinates brute force and stealth modes of infection in the plant pathogen *Pectobacterium atrosepticum* . PLoS Pathog 4: e1000093 doi:10.1371/journal.ppat.1000093 18566662 10.1371/journal.ppat.1000093 PMC2413422

[28] Datsenko KA, Wanner BL (2000) One-step inactivation of chromosomal genes in *Escherichia coli* K-12 using PCR products. Proc Natl Acad Sci U S A 97: 6640–6645. 10829079 10.1073/pnas.120163297 PMC18686

[29] Labrie SJ, Moineau S (2007) Abortive infection mechanisms and prophage sequences significantly influence the genetic makeup of emerging lytic lactococcal phages. J Bacteriol 189: 1482–1487. 17041060 10.1128/JB.01111-06 PMC1797345

[30] Hill C, Miller LA, Klaenhammer TR (1991) *In vivo* genetic exchange of a functional domain from a type II A methylase between lactococcal plasmid pTR2030 and a virulent bacteriophage. J Bacteriol 173: 4363–4370. 1906061 10.1128/jb.173.14.4363-4370.1991 PMC208097

[31] Weinberg Z, Perreault J, Meyer MM, Breaker RR (2009) Exceptional structured noncoding RNAs revealed by bacterial metagenome analysis. Nature 462: 656–659. 19956260 10.1038/nature08586 PMC4140389

[32] Roberts F, Allison GE, Verma NK (2007) Transcription-termination-mediated immunity and its prevention in bacteriophage SfV of *Shigella flexneri* . J Gen Virol 88: 3187–3197. 17947546 10.1099/vir.0.83062-0

[33] Petty NK, Toribio AL, Goulding D, Foulds I, Thomson N, Dougan G, Salmond GPC (2007) A generalized transducing phage for the murine pathogen *Citrobacter rodentium* . Microbiology 153: 2984–2988. 17768241 10.1099/mic.0.2007/008888-0 PMC2652034

[34] Monson R, Foulds I, Foweraker J, Welch M, Salmond GPC (2011) The *Pseudomonas aeruginosa* generalized transducing phage phiPA3 is a new member of the phiKZ-like group of ‘jumbo’ phages, and infects model laboratory strains and clinical isolates from cystic fibrosis patients. Microbiology 157: 859–867. 21163841 10.1099/mic.0.044701-0

[35] Haaber J, Moineau S, Hammer K (2009) Activation and transfer of the chromosomal phage resistance mechanism AbiV in *Lactococcus lactis* . Appl Environ Microbiol 75: 3358–3361. 19286782 10.1128/AEM.02538-08 PMC2681626

[36] Klaenhammer TR, Sanozky RB (1985) Conjugal transfer from *Streptococcus lactis* ME2 of plasmids encoding phage resistance, nisin resistance and lactose-fermenting ability: evidence for a high-frequency conjugative plasmid responsible for abortive infection of virulent bacteriophage. J Gen Microbiol 131: 1531–1541. 3930657 10.1099/00221287-131-6-1531

[37] Petty NK, Foulds IJ, Pradel E, Ewbank JJ, Salmond GP (2006) A generalized transducing phage (ΦIF3) for the genomically sequenced *Serratia marcescens* strain Db11: a tool for functional genomics of an opportunistic human pathogen. Microbiology 152: 1701–1708. 16735733 10.1099/mic.0.28712-0

[38] Delcher AL, Bratke KA, Powers EC, Salzberg SL (2007) Identifying bacterial genes and endosymbiont DNA with Glimmer. Bioinformatics 23: 673–679. 17237039 10.1093/bioinformatics/btm009 PMC2387122

[39] Altschul SF, Gish W, Miller W, Myers EW, Lipman DJ (1990) Basic local alignment search tool. J Mol Biol 215: 403–410. 2231712 10.1016/S0022-2836(05)80360-2

[40] Lowe TM, Eddy SR (1997) tRNAscan-SE: a program for improved detection of transfer RNA genes in genomic sequence. Nucl Acids Res 25: 955–964. 9023104 10.1093/nar/25.5.955 PMC146525

[41] Suzek BE, Ermolaeva MD, Schreiber M, Salzberg SL (2001) A probabilistic method for identifying start codons in bacterial genomes. Bioinformatics 17: 1123–1130. 11751220 10.1093/bioinformatics/17.12.1123

[42] Rutherford K, Parkhill J, Crook J, Horsnell T, Rice P, et al (2000) Artemis: sequence visualization and annotation. Bioinformatics 16: 944–945. 11120685 10.1093/bioinformatics/16.10.944

[43] Grant JR, Stothard P (2008) The CGView Server: a comparative genomics tool for circular genomes. Nucl Acids Res 36: W181–184. 18411202 10.1093/nar/gkn179 PMC2447734

[44] Fineran PC, Everson L, Slater H, Salmond GP (2005) A GntR family transcriptional regulator (PigT) controls gluconate-mediated repression and defines a new, independent pathway for regulation of the tripyrrole antibiotic, prodigiosin, in Serratia. Microbiology 151: 3833–3845. 16339930 10.1099/mic.0.28251-0

[45] Przybilski R, Graf S, Lescoute A, Nellen W, Westhof E, et al (2005) Functional hammerhead ribozymes naturally encoded in the genome of *Arabidopsis thaliana* . Plant Cell 17: 1877–1885. 15937227 10.1105/tpc.105.032730 PMC1167538

[46] Przybilski R, Richter C, Gristwood T, Clulow JS, Vercoe RB, et al (2011) Csy4 is responsible for CRISPR RNA processing in *Pectobacterium atrosepticum* . RNA Biol 8: 517–528. 21519197 10.4161/rna.8.3.15190

