# Supplementary figures and images for: Viral Evasion of a Bacterial Suicide System by RNA–Based Molecular Mimicry Enables Infectious Altruism
Source: PLoS Genet. 2012 Oct 18;8(10):e1003023. doi: 10.1371/journal.pgen.1003023 (PMC3475682; doi:10.1371/journal.pgen.1003023)

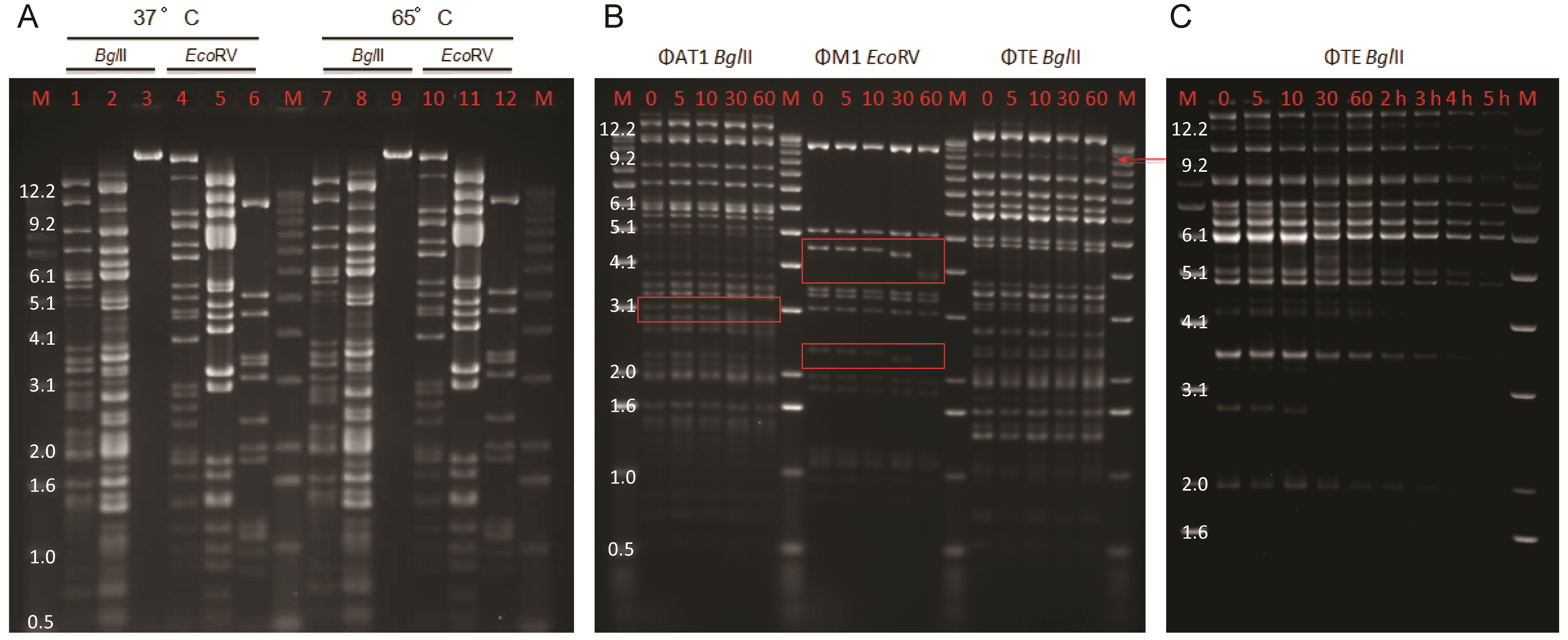

Supplement: Figure S1 — Characterisation of the ΦTE genome (A–C) Restriction digestion of ΦAT1, ΦM1 and ΦTE genomic DNAs. (A) Identification of cos sites. Lanes 1, 4, 7, 10: ΦAT1; lanes 2, 5, 8, 11: ΦTE; lanes 3, 6, 9, 12: ΦM1. Lanes 7–12 are the same as lanes 1–6 except digestion reactions were heated to 65°C for 20 min before loading to attempt to identify cos sites; none were evident. (B) Phage genomic DNA was treated with a Bal-31 exonuclease time course before restriction digestion to determine if the genome was circularly permuted. Numbers indicate the length of Bal-31 treatment (min). Specific bands were observed to be preferentially degraded by Bal-31 treatment of ΦAT1 and ΦM1, suggesting these two phages are not circularly permuted (red boxes). The sub-molar fragment in the ΦTE digest could represent the pac fragment, and is indicated with a red arrow. (C) ΦTE is circularly permuted. ΦTE digest, as in (B), but with longer incubation, showing that all restriction fragments are lost following Bal-31 treatment, proving that ΦTE has a circularly permuted genome. ‘M’ indicates DNA size markers in Kb (1 Kb ladder, Invitrogen). (TIF) [file pgen.1003023.s001.tif]
