# Supplementary material for: Viral Evasion of a Bacterial Suicide System by RNA–Based Molecular Mimicry Enables Infectious Altruism
Source: PLoS Genet. 2012 Oct 18;8(10):e1003023. doi: 10.1371/journal.pgen.1003023 (PMC3475682; doi:10.1371/journal.pgen.1003023)
Supplement: Table S1 — Transposon insertion sites within ΦTE-resistant strains. (DOCX) [file pgen.1003023.s002.docx]

| **Table S1.** Transposon insertion sites within ΦTE-resistant strains | | | | |
| --- | --- | --- | --- | --- |
|  |  |  |  |  |
|  |  |  |  |  |
| Mutant strain | Disrupted gene |  | First base of insertion^a^ |  |
|  |  |  |  |  |
| TER2 | *flgN* |  | 1949373 |  |
| TER7 | *flhA* |  | 1948418 |  |
| TER9 | *ECA1732* |  | 1978027 |  |
| TER19 | *flgH* |  | 1955827 |  |
| TER21 | *fliR* |  | 1962168 |  |
| TER22 | *ECA1732* |  | 1978826 |  |
| TER23 | *flgH* |  | 1956068 |  |
|  |  |  |  |  |
|  |  |  |  |  |
| **a.** Genome co-ordinates correspond to the *Pectobacterium atrosepticum* SCRI1043 genome sequence, accession number NC_004547 | | | | |
