# Supplementary material for: Viral Evasion of a Bacterial Suicide System by RNA–Based Molecular Mimicry Enables Infectious Altruism
Source: PLoS Genet. 2012 Oct 18;8(10):e1003023. doi: 10.1371/journal.pgen.1003023 (PMC3475682; doi:10.1371/journal.pgen.1003023)
Supplement: Table S2 — Details of ORFs, tRNAs and ncRNA within ΦTE wild-type genome. (DOCX) [file pgen.1003023.s003.docx]

| **Table S2.** Details of ORFs, tRNAs and ncRNA within ΦTE wild type genome | | | | | | | |
| --- | --- | --- | --- | --- | --- | --- | --- |
| Gene |  | BLASTp hit | E value | Nucleotides (includes stop codon of ORF) | Amino acids | Start codon | SD sequence |
| phiTE_001 |  | ref\|NP_945017.1\| Putative HNH endonuclease [Enterobacteria phage Felix 01] | 2.0E-36 | 504 | 167 | ATG | NONE |
| phiTE_002 |  | gb\|ABI79279.1\| hypothetical protein [*Escherichia* phage rv5] | 4.0E-30 | 1227 | 408 | ATG | AGGAG |
| phiTE_003 |  | No significant similarity found |  | 390 | 129 | GTG | GGGAT |
| phiTE_004 |  | No significant similarity found |  | 693 | 230 | ATG | AGGAG |
| phiTE_005 |  | No significant similarity found |  | 216 | 71 | ATG | AGGAG |
| phiTE_006 |  | gb\|ADP02413.1\| conserved hypothetical protein [*Salmonella* phage PVP-SE1] | 3.0E-31 | 351 | 116 | ATG | NONE |
| phiTE_007 |  | ref\|YP_358789.1\| putative HNH endonuclease [*Lactobacillus* phage Lc-Nu] | 2.0E-06 | 384 | 127 | ATG | GGGAG |
| phiTE_008 |  | ref\|YP_002003725.1\| DNA polymerase [*Escherichia* phage rv5] | 4.0E-52 | 336 | 111 | ATG | AGGGG |
| phiTE_009 |  | ref\|ZP_01235487.1\| hypothetical protein VAS14_01911 [*Vibrio angustum* S14] | 7.0E-07 | 450 | 149 | ATG | AGGAG |
| phiTE_010 |  | ref\|YP_002003725.1\| DNA polymerase [*Escherichia* phage rv5] | 0.0E+00 | 1998 | 665 | ATG | GGGAG |
| phiTE_011 |  | ref\|YP_002330023.1\| predicted HNH endonuclease [*Escherichia coli* O127:H6 str. E2348/69] | 2.0E-21 | 513 | 170 | ATG | AGGAG |
| phiTE_012 |  | No significant similarity found |  | 213 | 70 | ATG | TGGAG |
| phiTE_013 |  | gb\|ADP02603.1\| hypothetical protein [*Salmonella* phage PVP-SE1] | 4.0E-21 | 471 | 156 | ATG | CGGAG |
| phiTE_014 |  | No significant similarity found |  | 402 | 133 | ATG | GGGAG |
| phiTE_015 |  | ref\|NP_061537.1\| Orf41 [*Pseudomonas* phage D3] | 2.0E-05 | 285 | 94 | ATG | GGGAG |
| phiTE_016 |  | No significant similarity found |  | 267 | 88 | ATG | AGGAA |
| phiTE_017 |  | No significant similarity found |  | 201 | 66 | ATG | CGGAG |
| phiTE_018 |  | emb\|CAZ39580.1\| hypothetical protein [*Erwinia* phage phiAT1] | 2.0E-82 | 369 | 122 | ATG | AGGAG |
|  |  | ref\|NP_059615.1\| NinX [Enterobacteria phage P22] | 2.0E-15 |  |  |  |  |
| phiTE_019 |  | ref\|ZP_01963391.1\| hypothetical protein RUMOBE_01107 [*Ruminococcus obeum* ATCC 29174] | 4.0E-21 | 1206 | 401 | ATG | ACGAG |
| phiTE_020 |  | No significant similarity found |  | 273 | 90 | GTG | GCGAG |
| phiTE_021 |  | gb\|AEH03770.1\| hypothetical protein [*Pseudomonas* phage PhiPA3] | 8.0E-52 | 1170 | 389 | ATG | TGGGG |
| phiTE_022 |  | ref\|YP_002922206.1\| hypothetical protein EpJSE_00134 [Enterobacteria phage JSE] | 2.0E-10 | 723 | 240 | ATG | AGAAG |
| phiTE_023 |  | ref\|NP_775217.1\| DNA primase/helicase [*Pseudomonas* phage PaP3] | 2.0E-57 | 2079 | 692 | GTG | AGGGG |
| phiTE_024 |  | No significant similarity found |  | 168 | 55 | ATG | GGGGG |
| phiTE_025 |  | gb\|ADP02403.1\| hypothetical protein [*Salmonella* phage PVP-SE1 | 2.0E-03 | 276 | 91 | ATG | AGGGG |
| phiTE_026 |  | No significant similarity found |  | 192 | 63 | ATG | TGGAG |
| phiTE_027 |  | No significant similarity found |  | 216 | 71 | GTG | TGGAG |
| phiTE_028 |  | No significant similarity found |  | 186 | 61 | ATG | GGGAG |
| phiTE_029 |  | No significant similarity found |  | 591 | 196 | TTG | NONE |
| phiTE_030 |  | gb\|ADP02399.1\| putative helicase [*Salmonella* phage PVP-SE1] | 3.0E-89 | 2472 | 823 | ATG | AGGCG |
| phiTE_031 |  | No significant similarity found |  | 186 | 61 | ATG | GGGAG |
| phiTE_032 |  | No significant similarity found |  | 198 | 65 | ATG | AGGAG |
| phiTE_033 |  | gb\|ADP02639.1\| cell wall hydrolase SleB [*Salmonella* phage PVP-SE1] | 5.0E-49 | 531 | 176 | ATG | AGGAG |
| phiTE_034 |  | No significant similarity found |  | 381 | 126 | ATG | AGGAG |
| phiTE_035 |  | gb\|ADP02628.1\| putative phosphatase [*Salmonella* phage PVP-SE1] | 2.0E-33 | 429 | 142 | ATG | GGGAG |
| phiTE_036 |  | No significant similarity found |  | 213 | 70 | GTG | AGTAG |
| phiTE_037 |  | No significant similarity found |  | 192 | 63 | TTG | ATGAG |
| phiTE_038 |  | No significant similarity found |  | 231 | 76 | ATG | TGGAG |
| phiTE_039 |  | No significant similarity found |  | 99 | 32 | GTG | AGGAG |
| phiTE_040 |  | No significant similarity found |  | 147 | 48 | GTG | AGGTG |
| phiTE_041 |  | No significant similarity found |  | 171 | 56 | ATG | TGGAG |
| phiTE_042 |  | No significant similarity found |  | 450 | 149 | ATG | AGAGG |
| phiTE_043 |  | No significant similarity found |  | 189 | 62 | ATG | GGGAG |
| phiTE_044 |  | No significant similarity found |  | 324 | 107 | ATG | TGGAG |
| phiTE_045 |  | No significant similarity found |  | 267 | 88 | GTG | NONE |
| phiTE_046 |  | No significant similarity found |  | 345 | 114 | ATG | TGGAG |
| phiTE_047 |  | emb\|CCE26178.1\| g154 [*Yersinia* phage phiR1-37] | 5.0E-17 | 732 | 243 | ATG | AGGTG |
| phiTE_048 |  | No significant similarity found |  | 207 | 68 | ATG | AGGAG |
| phiTE_049 |  | No significant similarity found |  | 246 | 81 | ATG | AGGAG |
| phiTE_050 |  | No significant similarity found |  | 174 | 57 | ATG | CGGGG |
| phiTE_051 |  | ref\|NP_944949.1\| hypothetical protein Felix01p170 [Enterobacteria phage Felix 01] | 4.0E-07 | 267 | 88 | ATG | TGGAG |
| phiTE_052 |  | No significant similarity found |  | 234 | 77 | ATG | CGGAG |
| phiTE_053 |  | No significant similarity found |  | 198 | 65 | TTG | ATGAG |
| phiTE_054 |  | No significant similarity found |  | 297 | 98 | GTG | GGGAG |
| phiTE_055 |  | gb\|ADP02616.1\| conserved hypothetical protein [*Salmonella* phage PVP-SE1] | 8.0E-47 | 1242 | 413 | ATG | AGGAG |
|  |  | ref\|YP_004301226.1\| putative metal dependent phosphohydrolase [*Aeromonas* phage 65] | 5.0E-25 |  |  |  |  |
| phiTE_056 |  | No significant similarity found |  | 162 | 53 | ATG | AGGAT |
| phiTE_057 |  | No significant similarity found |  | 192 | 63 | GTG | AGGAG |
| phiTE_058 |  | gb\|ADP02621.1\| MoxR ATPase [*Salmonella* phage PVP-SE1] | 2.0E-91 | 1239 | 412 | ATG | AGGAG |
| phiTE_059 |  | gb\|ADQ12725.1\| putative HNH endonuclease [*Acinetobacter* phage phiAB1] | 6.0E-21 | 513 | 170 | ATG | AGGAT |
| phiTE_060 |  | gb\|ADP02612.1\| conserved hypothetical protein [*Salmonella* phage PVP-SE1] | 1.0E-113 | 1551 | 516 | ATG | CGGAG |
| phiTE_061 |  | No significant similarity found |  | 381 | 126 | ATG | AGGAG |
| phiTE_062 |  | No significant similarity found |  | 168 | 55 | GTG | AGGGG |
| phiTE_063 |  | dbj\|BAE47722.1\| conserved hypothetical protein [*Clostridium* phage c-st] | 6.0E-04 | 255 | 84 | GTG | CGGAG |
| phiTE_064 |  | ref\|ZP_00208964.1\| hypothetical protein Magn03005705 [*Magnetospirillum magnetotacticum* MS-1] | 4.0E-05 | 243 | 80 | ATG | AGGAG |
| phiTE_065 |  | No significant similarity found |  | 162 | 53 | TTG | AGGAG |
| phiTE_066 |  | No significant similarity found |  | 123 | 40 | ATG | TGGAG |
| phiTE_067 |  | No significant similarity found |  | 321 | 106 | ATG | GGGGG |
| phiTE_068 |  | No significant similarity found |  | 279 | 92 | ATG | CGGAG |
| phiTE_069 |  | ref\|NP_640228.1\| hypothetical protein Rts1_267 [*Proteus vulgaris*] | 5.0E-10 | 429 | 142 | ATG | AAGAG |
| phiTE_070 |  | ref\|ZP_03827032.1\| hypo prot PcarbP_10457 [*Pecto. carotovorum* subsp. *brasiliensis* PBR1692] | 1.0E-13 | 306 | 101 | ATG | GGGAG |
| phiTE_071 |  | No significant similarity found |  | 114 | 37 | TTG | NONE |
| phiTE_072 |  | ref\|ZP_06636728.1\| conserved hypothetical protein [*Serratia odorifera* DSM 4582] | 1.0E-37 | 549 | 182 | ATG | GGGGG |
| phiTE_073 |  | No significant similarity found |  | 168 | 55 | ATG | GGGAG |
| phiTE_074 |  | ref\|YP_003358575.1\| hypothetical protein [*Shigella* phage phiSboM-AG3] | 2.0E-11 | 192 | 63 | ATG | TGGAG |
| phiTE_075 |  | No significant similarity found |  | 231 | 76 | ATG | TGGAG |
| phiTE_076 |  | No significant similarity found |  | 282 | 93 | ATG | TGGAG |
| phiTE_077 |  | No significant similarity found |  | 258 | 85 | ATG | NONE |
| phiTE_078 |  | No significant similarity found |  | 837 | 278 | ATG | AGGAG |
| phiTE_079 |  | No significant similarity found |  | 432 | 143 | GTG | NONE |
| phiTE_080 |  | No significant similarity found |  | 333 | 110 | ATG | AGGAG |
| phiTE_081 |  | ref\|YP_002157443.1\| hypothetical protein VFMJ11_B0081 [*Vibrio fischeri* MJ11] | 2.0E-32 | 519 | 172 | TTG | AGAAG |
| phiTE_082 |  | No significant similarity found |  | 345 | 114 | ATG | TGGAG |
| phiTE_083 |  | gb\|ACY75978.1\| predicted protein [*Prochlorococcus* phage P-SSM2] | 1.0E-17 | 225 | 74 | ATG | ACGAG |
| phiTE_084 |  | No significant similarity found |  | 468 | 155 | TTG | NONE |
| phiTE_085 |  | No significant similarity found |  | 210 | 69 | ATG | AAGAG |
| phiTE_086 |  | ref\|YP_002003692.1\| hypothetical protein rv5_gp190 [*Escherichia* phage rv5] | 2.0E-18 | 462 | 153 | TTG | GGGAT |
| phiTE_087 |  | No significant similarity found |  | 312 | 103 | ATG | TGGGG |
| phiTE_088 |  | No significant similarity found |  | 240 | 79 | ATG | AGGGG |
| phiTE_089 |  | No significant similarity found |  | 438 | 145 | ATG | GAGAG |
| phiTE_090 |  | gb\|ADP02584.1\| hypothetical protein [*Salmonella* phage PVP-SE1] | 2.0E-25 | 258 | 85 | ATG | ACGAG |
| phiTE_091 |  | gb\|EHC91128.1\| Phage EaA protein [*Salmonella* *ent.* subsp. *enterica* serovar Senftenberg str. A4-543] | 2.0E-03 | 627 | 208 | ATG | AGGAT |
| phiTE_092 |  | No significant similarity found |  | 123 | 40 | ATG | CGGGG |
| phiTE_093 |  | No significant similarity found |  | 387 | 128 | ATG | CGGGG |
| phiTE_094 |  | No significant similarity found |  | 525 | 174 | GTG | AGGAT |
| phiTE_095 |  | No significant similarity found |  | 264 | 87 | ATG | NONE |
| phiTE_096 |  | No significant similarity found |  | 168 | 55 | ATG | TGGAG |
| phiTE_097 |  | ref\|YP_453589.1\| HNH endonuclease family protein [*Xanthomonas* phage OP1] | 3.0E-16 | 501 | 166 | ATG | AGTAG |
| phiTE_098 |  | No significant similarity found |  | 213 | 70 | ATG | AGGGG |
| phiTE_099 |  | No significant similarity found |  | 321 | 106 | ATG | GGGGG |
| phiTE_100 |  | No significant similarity found |  | 324 | 107 | GTG | AGGAG |
| phiTE_101 |  | No significant similarity found |  | 336 | 111 | ATG | CGGGG |
| phiTE_102 |  | No significant similarity found |  | 177 | 58 | TTG | AGGGG |
| phiTE_103 |  | No significant similarity found |  | 225 | 74 | ATG | AGAGG |
| phiTE_104 |  | No significant similarity found |  | 147 | 48 | ATG | GGGGG |
| phiTE_105 |  | No significant similarity found |  | 252 | 83 | ATG | CGGAG |
| phiTE_106 |  | No significant similarity found |  | 471 | 156 | ATG | AGGGG |
| phiTE_107 |  | ref\|NP_049833.1\| Cd.5 hypothetical protein [Enterobacteria phage T4] | 7.0E-04 | 249 | 82 | TTG | TGGGG |
| phiTE_108 |  | ref\|NP_848253.1\| Ead-like protein [Enterobacteria phage epsilon15] | 3.0E-10 | 390 | 129 | ATG | GGGAG |
| phiTE_109 |  | ref\|YP_006372.1\| gp17 [Enterobacteria phage ST104] | 3.0E-20 | 333 | 110 | ATG | AGGAG |
|  |  | dbj\|BAF80755.1\| superinfection exclusion protein [Enterobacteria phage P22] | 2.0E-19 |  |  |  |  |
| phiTE_110 |  | No significant similarity found |  | 183 | 60 | TTG | AGGAG |
| phiTE_111 |  | No significant similarity found |  | 270 | 89 | ATG | GGGAG |
| phiTE_112 |  | No significant similarity found |  | 600 | 199 | ATG | AGGGG |
| phiTE_113 |  | No significant similarity found |  | 513 | 170 | ATG | CGGAG |
| phiTE_114 |  | No significant similarity found |  | 231 | 76 | GTG | AGGAG |
| phiTE_115 |  | No significant similarity found |  | 330 | 109 | ATG | AGGAG |
| phiTE_116 |  | No significant similarity found |  | 228 | 75 | GTG | GGGAG |
| phiTE_117 |  | ref\|YP_004009882.1\| hypothetical protein CC31p024 [Enterobacteria phage CC31] | 3.0E-05 | 240 | 79 | ATG | TGGAG |
| phiTE_118 |  | No significant similarity found |  | 171 | 56 | ATG | ACGAG |
| phiTE_119 |  | No significant similarity found |  | 501 | 166 | TTG | TGGAG |
| phiTE_120 |  | ref\|YP_004009936.1\| hypothetical protein CC31p078 [Enterobacteria phage CC31] | 1.0E-36 | 339 | 112 | ATG | CGGAG |
| phiTE_121 |  | No significant similarity found |  | 210 | 69 | GTG | AGGAG |
| phiTE_122 |  | No significant similarity found |  | 321 | 106 | GTG | GGGAG |
| phiTE_123 |  | No significant similarity found |  | 450 | 149 | ATG | TGAAG |
| phiTE_124 |  | No significant similarity found |  | 402 | 133 | GTG | TGGAG |
| phiTE_125 |  | No significant similarity found |  | 432 | 143 | TTG | AGGCG |
| phiTE_126 |  | No significant similarity found |  | 444 | 147 | ATG | GGGAG |
| phiTE_127 |  | ref\|YP_004009740.1\| hypothetical protein Acj61p123 [*Acinetobacter* phage Acj61] | 9.0E-19 | 594 | 197 | ATG | GGGGG |
| phiTE_128 |  | gb\|AEO93545.1\| gp286 [Bacillus phage G] | 7.0E-08 | 261 | 86 | ATG | AGGAG |
| phiTE_129 |  | No significant similarity found |  | 477 | 158 | ATG | GGGAG |
| phiTE_130 |  | gb\|ADP02562.1\| conserved hypothetical protein [*Salmonella* phage PVP-SE1] | 1.0E-06 | 174 | 57 | TTG | AGAGG |
| phiTE_131 |  | gb\|ADP02562.1\| conserved hypothetical protein [*Salmonella* phage PVP-SE1] | 3.0E-12 | 120 | 39 | GTG | GGCAG |
| phiTE_132 |  | gb\|ADP02562.1\| putative nucleotidyltransferase [*Salmonella* phage PVP-SE1] | 8.0E-91 | 798 | 265 | ATG | TGGAG |
| phiTE_133 |  | No significant similarity found |  | 402 | 133 | ATG | AGGAG |
| phiTE_134 |  | ref\|YP_002891059.1\| hypothetical protein peH4H_0016 [*Escherichia coli*] | 2.0E-04 | 399 | 132 | ATG | AAGGG |
| phiTE_135 |  | No significant similarity found |  | 300 | 99 | ATG | AGGGG |
| phiTE_136 |  | gb\|ADP02555.1\| tRNA nucleotidyl transferase [*Salmonella* phage PVP-SE1] | 3.0E-115 | 1326 | 441 | ATG | AGGAG |
| phiTE_137 |  | emb\|CBX44478.1\| HNH endonuclease [*Erwinia* phage phiEa1H] | 3.0E-12 | 510 | 169 | ATG | AGGAG |
| phiTE_138 |  | No significant similarity found |  | 333 | 110 | ATG | AGGAG |
| phiTE_139 |  | ref\|YP_002456113.1\| putative HNH endonuclease [*Erwinia* phage phiEa21-4] | 2.0E-21 | 432 | 143 | TTG | GGGAG |
| phiTE_140 |  | gb\|ADP02548.1\| putative DNA methyltransferase, conserved hypo. protein [*Salmonella* phage PVP-SE1] | 2.0E-74 | 540 | 179 | ATG | GGGGG |
| phiTE_141 |  | gb\|ADP02547.1\| ClpP ATP-dependent protease subunit [*Salmonella* phage PVP-SE1] | 2.0E-23 | 903 | 300 | ATG | AGGGG |
| phiTE_142 |  | gb\|ADP02546.1\| hypothetical protein [*Salmonella* phage PVP-SE1] | 5.0E-09 | 477 | 158 | ATG | GGGAG |
| phiTE_143 |  | No significant similarity found |  | 318 | 105 | ATG | GGGAG |
| phiTE_144 |  | gb\|ADP02538.1\| anaerobic NTP reductase, small subunit [*Salmonella* phage PVP-SE1] | 2.0E-86 | 471 | 156 | ATG | AGGTG |
| phiTE_145 |  | gb\|ADP02537.1\| anaerobic ribonucleoside-triphosphate reductase [*Salmonella* phage PVP-SE1] | 0.0E+00 | 2037 | 678 | ATG | AGCGG |
| phiTE_146 |  | ref\|YP_001671805.1\| phosphate starvation-inducible phoH-like prot. [Enterobacteria phage phiEco32] | 2.0E-105 | 750 | 249 | ATG | AGGAG |
| phiTE_147 |  | emb\|CAJ28446.1\| endolysin [*Yersinia* phage PY100] | 1.0E-63 | 384 | 127 | ATG | AGGAG |
| phiTE_148 |  | No significant similarity found |  | 414 | 137 | ATG | AGGCG |
| phiTE_149 |  | ref\|YP_003060657.1\| glutaredoxin [*Hirschia baltica* ATCC 49814] | 1.0E-15 | 288 | 95 | ATG | TGGAG |
| phiTE_150 |  | No significant similarity found |  | 225 | 74 | ATG | TGGAG |
| phiTE_151 |  | No significant similarity found |  | 222 | 73 | ATG | AGGAG |
| phiTE_152 |  | ref\|ZP_08102728.1\| ribonucleotide-diphosphate reductase subunit beta [*Vibrio sinaloensis* DSM 21326] | 1.0E-86 | 2064 | 687 | ATG | AGGAG |
| phiTE_153 |  | No significant similarity found |  | 171 | 56 | ATG | AGGAG |
| phiTE_154 |  | gb\|ADX88246.1\| ribonucleoside triphosphate reductase, alpha chain [*Vibrio* phage ICP1_2006_D] | 8.0E-178 | 2352 | 783 | ATG | GGGAG |
| phiTE_155 |  | ref\|YP_862450.1\| HNH endonuclease family protein [*Gramella forsetii* KT0803] | 5.0E-21 | 492 | 163 | ATG | AGGGG |
| phiTE_156 |  | ref\|ZP_04716875.1\| ribonucleotide-diphosphate reductase subunit alpha [*Alter. macleodii* ATCC 27126] | 7.0E-93 | 762 | 253 | ATG | CGGAG |
| phiTE_157 |  | gb\|ADP02532.1\| conserved hypothetical protein [*Salmonella* phage PVP-SE1] | 1.0E-11 | 231 | 76 | ATG | AGGAG |
| phiTE_158 |  | gb\|ADP02531.1\| conserved hypothetical protein [*Salmonella* phage PVP-SE1] | 5.0E-23 | 495 | 164 | ATG | AGGAG |
| phiTE_159 |  | ref\|YP_004782449.1\| thymidylate synthase [*Salmonella* phage 7-11] | 6.0E-52 | 681 | 226 | ATG | AGGAG |
| phiTE_160 |  | ref\|ZP_01994802.1\| hypothetical protein DORLON_00791 [Dorea longicatena DSM 13814] | 1.0E-35 | 684 | 227 | ATG | AGGAG |
| phiTE_161 |  | gb\|ADP02527.1\| conserved hypothetical protein [*Salmonella* phage PVP-SE1] | 5.0E-10 | 204 | 67 | TTG | GGCAG |
| phiTE_162 |  | gb\|ADP02526.1\| conserved hypothetical protein [*Salmonella* phage PVP-SE1] | 6.0E-69 | 612 | 203 | ATG | AGGAG |
| phiTE_163 |  | ref\|YP_002003601.1\| hypothetical protein rv5_gp099 [*Escherichia* phage rv5] | 6.0E-43 | 657 | 218 | ATG | AGGAG |
| phiTE_164 |  | gb\|ADP02523.1\| conserved hypothetical protein [*Salmonella* phage PVP-SE1] | 2.0E-135 | 1068 | 355 | GTG | AGGGG |
|  |  | ref\|YP_453594.1\| putative DNA polymerase III epsilon subunit [*Xanthomonas* phage OP1] | 1.0E-32 |  |  |  |  |
| phiTE_165 |  | ref\|YP_001285569.1\| EndZ [Enterobacteria phage TLS] | 8.0E-32 | 537 | 178 | ATG | TTGAG |
| phiTE_166 |  | gb\|ADP02522.1\| conserved hypothetical protein [*Salmonella* phage PVP-SE1] | 2.0E-19 | 231 | 76 | ATG | TGGAG |
| phiTE_167 |  | gb\|ADP02521.1\| EndoVII packaging and recombination endonuclease [*Salmonella* phage PVP-SE1] | 1.0E-47 | 570 | 189 | GTG | TGGAG |
| phiTE_168 |  | No significant similarity found |  | 552 | 183 | ATG | AGCAG |
| phiTE_169 |  | gb\|ADP02520.1\| conserved hypothetical protein [*Salmonella* phage PVP-SE1] | 1.0E-43 | 624 | 207 | ATG | TGGAG |
| phiTE_170 |  | No significant similarity found |  | 333 | 110 | ATG | GGGGG |
| phiTE_171 |  | gb\|ADP02518.1\| exonuclease [*Salmonella* phage PVP-SE1] | 7.0E-178 | 1143 | 380 | ATG | AGAAG |
| phiTE_172 |  | ref\|YP_001742088.1\| putative endonuclease protein [*Salmonella* phage E1] | 4.0E-33 | 516 | 171 | ATG | AGAAG |
| phiTE_173 |  | gb\|ADP02517.1\| putative HNH endonuclease [*Salmonella* phage PVP-SE1] | 1.0E-46 | 504 | 167 | GTG | AGGAG |
| phiTE_174 |  | ref\|YP_453589.1\| HNH endonuclease family protein [*Xanthomonas* phage OP1] | 2.0E-15 | 513 | 170 | ATG | GGGTG |
| phiTE_175 |  | gb\|ADP02516.1\| conserved hypothetical protein [*Salmonella* phage PVP-SE1] | 7.0E-59 | 552 | 183 | TTG | GGTAG |
| phiTE_176 |  | No significant similarity found |  | 342 | 113 | ATG | AGGAT |
| phiTE_177 |  | ref\|YP_004010124.1\| hypothetical protein CC31p266 [Enterobacteria phage CC31] | 1.0E-49 | 342 | 113 | TTG | TGGAG |
| phiTE_178 |  | No significant similarity found |  | 264 | 87 | ATG | GGGCG |
| phiTE_179 |  | gb\|ADP02514.1\| hypothetical membrane protein [*Salmonella* phage PVP-SE1] | 4.0E-09 | 210 | 69 | ATG | AGGAG |
| phiTE_180 |  | ref\|YP_004009942.1\| hypothetical protein CC31p084 [Enterobacteria phage CC31] | 4.0E-04 | 474 | 157 | GTG | TGGAG |
| phiTE_181 |  | No significant similarity found |  | 285 | 94 | ATG | AGGAG |
| phiTE_182 |  | gb\|ADP02510.1\| conserved hypothetical protein [*Salmonella* phage PVP-SE1] | 6.0E-28 | 309 | 102 | ATG | AGGAG |
| phiTE_183 |  | gb\|AEM24705.1\| hypothetical protein [*Cronobacter* phage ES2] | 2.0E-16 | 504 | 167 | TTG | GGAAG |
| phiTE_184 |  | ref\|NP_891578.1\| gp39.2 conserved hypothetical protein [Enterobacteria phage RB49] | 1.0E-08 | 177 | 58 | ATG | TGGAG |
| phiTE_185 |  | ref\|YP_214420.1\| pyrophosphatase [*Prochlorococcus* phage P-SSM2] | 8.0E-18 | 363 | 120 | ATG | AGGAG |
| phiTE_186 |  | No significant similarity found |  | 441 | 146 | ATG | CGGAG |
| phiTE_187 |  | ref\|YP_004895209.1\| phage DNA ligase [*Salmonella* phage SFP10] | 2.0E-45 | 1380 | 459 | ATG | AGGAG |
| phiTE_188 |  | ref\|YP_239007.1\| hypothetical protein RB43ORF031c [Enterobacteria phage RB43] | 1.0E-26 | 399 | 132 | ATG | TGGAG |
| phiTE_189 |  | No significant similarity found |  | 300 | 99 | ATG | AGGGG |
| phiTE_190 |  | No significant similarity found |  | 117 | 38 | ATG | TGGAG |
| phiTE_191 |  | ref\|YP_002003520.1\| hypothetical protein rv5_gp018 [*Escherichia* phage rv5] | 1.0E-03 | 375 | 124 | ATG | AGAAG |
| phiTE_192 |  | gb\|ADP02494.1\| hypothetical protein [*Salmonella* phage PVP-SE1] | 2.0E-23 | 372 | 123 | GTG | AGGAG |
| phiTE_193 |  | gb\|ADP02492.1\| phosphoribosylpyrophosphate synthetase [*Salmonella* phage PVP-SE1] | 1.0E-59 | 891 | 296 | ATG | AGGAG |
| phiTE_194 |  | gb\|ADP02530.1\| HNH endonuclease [*Salmonella* phage PVP-SE1] | 3.0E-19 | 558 | 185 | GTG | TGGTG |
| phiTE_195 |  | gb\|ADP02491.1\| nicotinamide phosphoribosyl transferase [*Salmonella* phage PVP-SE1] | 8.0E-143 | 837 | 278 | ATG | AGGAG |
| phiTE_196 |  | ref\|YP_002300418.1\| gp31.2 [*Bacillus* phage SPO1] | 4.0E-24 | 585 | 194 | TTG | CGGAG |
| phiTE_197 |  | gb\|ADP02491.1\| nicotinamide phosphoribosyl transferase [*Salmonella* phage PVP-SE1] | 2.0E-111 | 999 | 332 | ATG | AGGAA |
| phiTE_198 |  | gb\|ADP02487.1\| conserved hypothetical protein [*Salmonella* phage PVP-SE1] | 3.0E-13 | 252 | 83 | ATG | NONE |
| phiTE_199 |  | No significant similarity found |  | 216 | 71 | GTG | AGGAG |
| phiTE_200 |  | No significant similarity found |  | 228 | 75 | ATG | AGGAG |
| phiTE_201 |  | ref\|YP_004009911.1\| hypothetical protein CC31p053 [Enterobacteria phage CC31] | 1.0E-04 | 207 | 68 | GTG | AGGGG |
| tRNA |  | tRNA-Cys, 105433-105506 bp, anticodon GCA at 105467-105469 bp |  |  |  |  |  |
| phiTE_202 |  | gb\|ADP02477.1\| conserved hypothetical protein [*Salmonella* phage PVP-SE1] | 9.0E-08 | 375 | 124 | ATG | GGGGG |
| tRNA |  | tRNA-Tyr, 106329-106407 bp, anticodon GTA at 106363-106365 bp |  |  |  |  |  |
| ncRNA |  | pseudo-ToxI, 1.5 repeats, 106714-106772 bp |  |  |  |  |  |
| phiTE_203 |  | No significant similarity found |  | 645 | 214 | ATG | AGGTG |
| phiTE_204 |  | gb\|ADP02473.1\| conserved hypothetical membrane protein [*Salmonella* phage PVP-SE1] | 1.0E-04 | 372 | 123 | ATG | AGGGG |
| phiTE_205 |  | gb\|ADP02472.1\| conserved hypothetical protein [*Salmonella* phage PVP-SE1] | 2.0E-04 | 315 | 104 | ATG | GGGGG |
| phiTE_206 |  | gb\|ADP02471.1\| terminase large subunit [*Salmonella* phage PVP-SE1] | 2.0E-53 | 384 | 127 | ATG | AGGAG |
| phiTE_207 |  | ref\|YP_002003568.1\| HNH homing endonuclease [*Escherichia* phage rv5] | 2.0E-34 | 678 | 225 | ATG | TGGAG |
| phiTE_208 |  | gb\|ADP02471.1\| terminase large subunit [*Salmonella* phage PVP-SE1] | 0.0E+00 | 1071 | 356 | ATG | GGGGG |
| phiTE_209 |  | gb\|ADP02470.1\| conserved hypothetical protein [*Salmonella* phage PVP-SE1] | 0.0E+00 | 1524 | 507 | GTG | AGGGG |
| phiTE_210 |  | emb\|CAZ39591.1\| hypothetical protein [*Erwinia* phage phiAT1] | 2.0E-48 | 576 | 191 | ATG | GGGAG |
| phiTE_211 |  | gb\|ADP02468.1\| conserved hypothetical protein [*Salmonella* phage PVP-SE1] | 2.0E-61 | 1110 | 369 | ATG | AAGAG |
| phiTE_212 |  | gb\|ADP02467.1\| head stabilization/decoration protein [*Salmonella* phage PVP-SE1] | 2.0E-13 | 450 | 149 | ATG | TGGAG |
| phiTE_213 |  | gb\|ADP02466.1\| putative major head protein [*Salmonella* phage PVP-SE1] | 2.0E-132 | 999 | 332 | ATG | AGGAG |
| phiTE_214 |  | gb\|ADP02465.1\| hypothetical protein [*Salmonella* phage PVP-SE1] | 1.0E-13 | 615 | 204 | ATG | AGGAG |
| phiTE_215 |  | ref\|YP_001956953.1\| virion structural protein [*Pseudomonas* phage 201phi2-1] | 4.0E-45 | 3300 | 1099 | ATG | AGGAG |
| phiTE_216 |  | emb\|CAZ39588.1\| hypothetical protein [*Erwinia* phage phiAT1] | 4.0E-75 | 651 | 216 | ATG | AGGAG |
| phiTE_217 |  | No significant similarity found |  | 495 | 164 | ATG | AGGAG |
| phiTE_218 |  | emb\|CAZ39589.1\| hypothetical protein [*Erwinia* phage phiAT1] | 4.0E-83 | 528 | 175 | ATG | TGGAG |
| phiTE_219 |  | gb\|ADP02459.1\| conserved hypothetical protein [*Salmonella* phage PVP-SE1] | 2.0E-38 | 462 | 153 | GTG | TGGGG |
| phiTE_220 |  | gb\|ADP02458.1\| conserved hypothetical protein [*Salmonella* phage PVP-SE1] | 1.0E-38 | 441 | 146 | ATG | AGGGG |
| phiTE_221 |  | gb\|ADP02457.1\| conserved hypothetical protein [*Salmonella* phage PVP-SE1] | 4.0E-55 | 708 | 235 | ATG | GGGAG |
| phiTE_222 |  | gb\|ADP02456.1\| structural protein [*Salmonella* phage PVP-SE1] | 6.0E-164 | 1422 | 473 | ATG | AGCAG |
| phiTE_223 |  | gb\|ADP02455.1\| structural protein [*Salmonella* phage PVP-SE1] | 6.0E-72 | 483 | 160 | ATG | AGGAG |
| phiTE_224 |  | gb\|ADP02454.1\| conserved hypothetical protein [*Salmonella* phage PVP-SE1] | 1.0E-47 | 477 | 158 | ATG | AGGAG |
| phiTE_225 |  | gb\|ADP02453.1\| conserved hypothetical protein [*Salmonella* phage PVP-SE1] | 2.0E-05 | 183 | 60 | ATG | GGAAG |
| phiTE_226 |  | No significant similarity found |  | 147 | 48 | ATG | TGGAG |
| phiTE_227 |  | ref\|YP_002922206.1\| hypothetical protein EpJSE_00134 [Enterobacteria phage JSE] | 1.0E-09 | 951 | 316 | ATG | TGGAG |
| phiTE_228 |  | ref\|YP_004306766.1\| putative tape measure protein [*Pseudomonas* phage KPP10] | 2.0E-21 | 2436 | 811 | ATG | AGGAG |
| phiTE_229 |  | gb\|ADP02451.1\| conserved hypothetical protein [*Salmonella* phage PVP-SE1] | 1.0E-53 | 855 | 284 | ATG | AGGAG |
| phiTE_230 |  | gb\|ADP02450.1\| conserved hypothetical protein [*Salmonella* phage PVP-SE1] | 3.0E-33 | 375 | 124 | TTG | GGGAG |
| phiTE_231 |  | emb\|CAZ39575.1\| hypothetical protein [*Erwinia* phage phiAT1] | 2.0E-169 | 993 | 330 | ATG | TGGAG |
| phiTE_232 |  | gb\|ADP02448.1\| putative baseplate assembly protein [*Salmonella* phage PVP-SE1] | 4.0E-65 | 744 | 247 | GTG | AGGAG |
| phiTE_233 |  | gb\|ADP02447.1\| conserved hypothetical protein [*Salmonella* phage PVP-SE1] | 1.0E-71 | 534 | 177 | ATG | AGGAG |
| phiTE_234 |  | gb\|ADP02443.1\| conserved hypothetical protein [*Salmonella* phage PVP-SE1] | 0.0E+00 | 1485 | 494 | ATG | AGGAG |
| phiTE_235 |  | gb\|ADP02442.1\| conserved hypothetical protein [*Salmonella* phage PVP-SE1] | 3.0E-72 | 645 | 214 | ATG | AGGAG |
| phiTE_236 |  | emb\|CAZ39578.1\| hypothetical protein [*Erwinia* phage phiAT1] | 1.0E-157 | 1665 | 554 | ATG | AGGAG |
|  |  | ref\|YP_001605990.1\| tail collar domain-containing protein [*Yersinia pestis* Angola] | 6.0E-30 |  |  |  |  |
| phiTE_237 |  | gb\|EGC05113.1\| caudovirales tail fibre assembly protein [*Escherichia fergusonii* B253] | 3.0E-19 | 516 | 171 | ATG | CGGGG |
| phiTE_238 |  | gb\|ADP02439.1\| conserved hypothetical membrane protein [*Salmonella* phage PVP-SE1] | 1.0E-26 | 339 | 112 | ATG | GGGAG |
| phiTE_239 |  | gb\|ADP02438.1\| hypothetical membrane protein [*Salmonella* phage PVP-SE1] | 4.0E-34 | 459 | 152 | ATG | TGGAG |
| phiTE_240 |  | gb\|ADP02437.1\| conserved hypothetical membrane protein [*Salmonella* phage PVP-SE1] | 4.0E-34 | 261 | 86 | ATG | AGGTG |
| phiTE_241 |  | gb\|ADP02436.1\| putative tail fiber protein [*Salmonella* phage PVP-SE1] | 1.0E-71 | 2382 | 793 | GTG | AGGAG |
| phiTE_242 |  | No significant similarity found |  | 1242 | 413 | ATG | TGGAG |
|  |  |  |  |  |  |  |  |
|  |  |  |  |  |  |  |  |
