# Supplementary material for: Viral Evasion of a Bacterial Suicide System by RNA–Based Molecular Mimicry Enables Infectious Altruism
Source: PLoS Genet. 2012 Oct 18;8(10):e1003023. doi: 10.1371/journal.pgen.1003023 (PMC3475682; doi:10.1371/journal.pgen.1003023)
Supplement: Table S3 — Full sequences and details of ΦTE escape loci. (DOCX) [file pgen.1003023.s004.docx]

| **Table S3.** Full sequences and details of ΦTE escape loci | | | | | |
| --- | --- | --- | --- | --- | --- |
|  |  |  |  |  |  |
|  |  |  |  |  |  |
| ΦTE phage | Full sequence including invariant 5′ and 3′ ends^a^ |  | Pattern of DNA repeats |  | Isolation Date |
|  |  |  |  |  |  |
| WT | ATTGACTCTATAGCTCAGCAGGTGATTCGCTACC**TT**AAGTGCGGGACGAAAATCCAGGTGATTCGCTACC**TTT**AAGTCGCAGGTCCGAGGTTCGA |  | 1.5 repeats; 1x 22 bp 'half' repeat (2T), 1x 37 bp repeat (3T) |  | 20th Jan 2010 |
| A and H | ATTGACTCTATAGCTCAGCAGGTGATTCGCTACC**TT**AAGTGCGGGACGAAAATCCAGGTGATTCGCTACC**TT**AAGTGCGGGACGAAAATCCAGGTGATTCGCTACC**TT**AAGTGCGGGACGAAAATCCAGGTGATTCGCTACC**TT**AAGTGCGGGACGAAAATCCAGGTGATTCGCTACC**TTT**AAGTCGCAGGTCCGAGGTTCGA |  | 4.5 repeats; 1x 22 bp 'half' repeat (2T), 3x 36 bp repeats (2T), 1x 37 bp repeat. Genome of H not sequenced |  | A isolated 20th Jan 2010, H isolated 22nd May 2010 |
| B, D and G | ATTGACTCTATAGCTCAGCAGGTGATTCGCTACC**TT**AAGTGCGGGACGAAAATCCAGGTGATTCGCTACC**TT**AAGTGCGGGACGAAAATCCAGGTGATTCGCTACC**TT**AAGTGCGGGACGAAAATCCAGGTGATTCGCTACC**TT**AAGTGCGGGACGAAAATCCAGGTGATTCGCTACC**TT**AAGTGCGGGACGAAAATCCAGGTGATTCGCTACC**TTT**AAGTCGCAGGTCCGAGGTTCGA |  | 5.5 repeats; 1x 22 bp 'half' repeat (2T), 4x 36 bp repeats (2T), 1x 37 bp repeat (3T). Genomes of each not sequenced |  | Both B and D isolated 20th Jan 2010, G isolated 22nd May 2010 |
| C and E | ATTGACTCTATAGCTCAGCAGGTGATTCGCTACC**TT**AAGTGCGGGACGAAAATCCAGGTGATTCGCTACC**TTT**AAGTGCGGGACGAAAATCCAGGTGATTCGCTACC**TTT**AAGTGCGGGACGAAAATCCAGGTGATTCGCTACC**TTT**AAGTGCGGGACGAAAATCCAGGTGATTCGCTACC**TTT**AAGTGCGGGACGAAAATCCAGGTGATTCGCTACC**TTT**AAGTCGCAGGTCCGAGGTTCGA |  | 5.5 repeats; 1x 22 bp 'half' repeat (2T), 5x 37 bp repeats (3T). Full genome sequences also identical |  | Both C and E isolated 20th Jan 2010 |
| F | ATTGACTCTATAGCTCAGCAGGTGATTCGCTACC**TTT**AAGTGCAGCTAGAAATTTAGGTGATTTGCTACC**TTT**AAGTGCAGCTAGAAATTCAGGTGATTTGCTACC**TTT**AAGTGCAGCTAGAAATTCAGGTGATTTGCTACC**TTT**AAGTGCAGCTAGAAATCCAGGTGATTCGCTACC**TTT**AAGTCGCAGGTCCGAGGTTCGA |  | 4 repeats; 1x 37 bp 'mosaic' repeat comprising first 18 bp from TE wt and 19 bp from the latter part of the first ToxI repeat (3T), 2x 36 bp ToxI repeats (3T), 1x 58 bp 'mosaic' repeat comprising 34 bp of a ToxI repeat (3T) and the latter 24 bp of a TE repeat (3T) |  | 20th Jan 2010 |
| I and J | ATTGACTCTATAGCTCAGCAGGTGATTCGCTACC**TT**AAGTGCGGGACGAAAATCCAGGTGATTCGCTACC**TT**AAGTGCGGGACGAAAATCCAGGTGATTCGCTACC**TTT**AAGTGCGGGACGAAAATCCAGGTGATTCGCTACC**TT**AAGTGCGGGACGAAAATCCAGGTGATTCGCTACC**TTT**AAGTGCGGGACGAAAATCCAGGTGATTCGCTACC**TTT**AAGTCGCAGGTCCGAGGTTCGA |  | 5.5 repeats; 1x 22 bp 'half' repeat (2T), 1x 36 bp repeat (2T), 1x 37 bp repeat (3T), 1x 36 bp repeat (2T), 2x 37 bp repeats (3T). Genomes of each not sequenced |  | Both I and J isolated 22nd May 2010 |
|  |  |  |  |  |  |
|  |  |  |  |  |  |
| **a.** Underlined sequences mark the invariant ends of the loci. Variant '2T' and '3T' sequences are in bold. | | | | | |
|  |  |  |  |  |  |
