# Supplementary material for: Viral Evasion of a Bacterial Suicide System by RNA–Based Molecular Mimicry Enables Infectious Altruism
Source: PLoS Genet. 2012 Oct 18;8(10):e1003023. doi: 10.1371/journal.pgen.1003023 (PMC3475682; doi:10.1371/journal.pgen.1003023)
Supplement: Table S4 — Bacterial strains and bacteriophages used in this study. (DOCX) [file pgen.1003023.s005.docx]

| **Table S4.** Bacterial strains and bacteriophages used in this study | | |
| --- | --- | --- |
| Organism | Relevant characteristic(s)^a^ | Reference or Source |
|  |  |  |
|  |  |  |
| *Bacterial strains* |  |  |
| *Escherichia coli* DH5α | K-12 strain; F^-^ Φ80*lacZ*ΔM15 Δ(*lacZYA-argF*) U169 *recA*1 *endA*1 *hsdR*17 (r_Κ_^-^, m_Κ_^+^) *phoA* *supE*44 λ^-^ *thi*-1 *gyrA*96 *relA*1 | Invitrogen |
| *Escherichia coli* β2163 | K-12 strain; F- RP4-2-Tc::Mu Δ*dapA*::(erm-pir), Em^R^, Km^R^ | [49] |
| *Pectobacterium atrosepticum* SCRI1043 | Wild type | [19] |
| SCC34 | Uncharacterised transposon mutant of Pba 1043, mucoid, ΦM1-resistant, Km^R^, derivative of Pba 1043 | This study |
| TER2 | *flgN*::Tn, ΦTE-resistant, Km^R^, derivative of Pba 1043 | This study |
| TER7 | *flhA*::Tn, ΦTE-resistant, Km^R^, derivative of Pba 1043 | This study |
| TER9 | *ECA1732*::Tn, ΦTE-resistant, Km^R^, derivative of Pba 1043 | This study |
| TER19 | *flgH*::Tn, ΦTE-resistant, Km^R^, derivative of Pba 1043 | This study |
| TER21 | *fliR*::Tn, ΦTE-resistant, Km^R^, derivative of Pba 1043 | This study |
| TER22 | *ECA1732*::Tn, ΦTE-resistant, Km^R^, derivative of Pba 1043 | This study |
| TER23 | *flgH*::Tn, ΦTE-resistant, Km^R^, derivative of Pba 1043 | This study |
| SCC14 | *virS*::Km^R^, derivative of Pba 1043 | [27] |
| SCC27 | *dsbA*::*uidA*, Cm^R^, derivative of Pba 1043 | [48] |
|  |  |  |
| *Bacteriophages* |  |  |
| ΦAT1 | *Myoviridae*, flagellatropic | [23] |
| ΦM1 | *Podoviridae*, LPS-dependent, generalised transducing phage of Pba 1043, ToxIN-sensitive | [20] |
| ΦS61 | ToxIN-sensitive | [12] |
| ΦTE | *Myoviridae*, flagellatropic, generalised transducing phage of Pba 1043, ToxIN-sensitive | This study, Milton sewage treatment plant |
| Escape phages ΦTE-A to J | *Myoviridae*, flagellatropic, generalised transducing phages of Pba 1043, ToxIN-insensitive | Selected on Pba 1043 (pTA46) |
|  |  |  |
|  | | |
| **a.** Cm^R^, chloramphenicol resistance; Em^R^, erythromycin resistance; Km^R^, kanamycin resistance. | | |
