# Supplementary material for: Viral Evasion of a Bacterial Suicide System by RNA–Based Molecular Mimicry Enables Infectious Altruism
Source: PLoS Genet. 2012 Oct 18;8(10):e1003023. doi: 10.1371/journal.pgen.1003023 (PMC3475682; doi:10.1371/journal.pgen.1003023)
Supplement: Table S5 — Primers used in this study. (DOCX) [file pgen.1003023.s006.docx]

| **Table S5.** Primers used in this study | | | |
| --- | --- | --- | --- |
| Primer | Sequence (5′-3′)^a^ | Description^b^ | Restriction site |
|  |  |  |  |
|  |  |  |  |
| KD01 | TTGGATCCGTTTTATCGACATTGTGAACC | FWD ToxIN Pba SCRI1039 pECA1039 | BamHI |
| MJ12 | TTTTAAGCTTCATATTTTTCTCGTAAAAAAGGCGACTATG | REV ToxI Pba SCRI1039 pECA1039 | HindIII |
| PF162 | GCAAATCACCTTAACTTAAGTATAC | REV ToxI promoter | - |
| PF168 | ACTTAAGTTAAGGTGATTTGCTACCTTTAAGTGCAGCTAGAAATTTAGGTGATTTACTACCTTTAAGTAATAAAGTAAAAGAGG | FWD 1.5 repeats ToxI and ToxN-FS, for overlap PCR with PF162 | - |
| PF185 | AAACAAATAGGGGTTCCG | FWD pQE-80L, for generating antitoxic inserts for pTA100. Use XhoI to cut resulting amplicon | - |
| PF217 | CGACGTAAAACGACGGCCAGT | FWD sequencing primer pBluescript | - |
| PF218 | GGAAACAGCTATGACCATG | REV sequencing primer pBluescript | - |
| PF221 | TTTTGAATTCGTTTTATCGACATTGTGAACC | FWD ToxI promoter | EcoRI |
| PF222 | TTTTCGGCCGTTTTAAGCTTTTTTGGATCCTTATTACTCGCCTTCTTCCGTATCCTC | REV ToxN | HindIII |
| TRB57 | TTTGAGCTCAAGGTGATTTGCTACCTTTAAG | FWD ToxI Pba SCRI1039 pECA1039 | SacI |
| TRB63 | TTTAAGCTTCTATTACTCGCCTTCTTCC | REV ToxN Pba SCRI1039 pECA1039 | HindIII |
| TRB164 | cggaagctttcaatcagg | FWD 13186 bp ΦTE | - |
| TRB165 | cggggtgtttaaggtaac | REV 14362 bp ΦTE | - |
| TRB166 | aaactggcgggatgtatg | FWD 39996 bp ΦTE | - |
| TRB167 | aataaagcggcgcttttc | REV 41005 bp ΦTE | - |
| TRB168 | acaattgcagatcacgcc | FWD 41489 bp ΦTE | - |
| TRB169 | ttgaaaccaacgtttgcc | REV 42620 bp ΦTE | - |
| TRB170 | cgcgaccaatagaaaagg | FWD 49323 bp ΦTE | - |
| TRB171 | ccttctgtgacaccttcc | REV 50420 bp ΦTE | - |
| TRB172 | tgcatcaatagatcctcc | FWD 56907 bp ΦTE | - |
| TRB173 | taaggttcttgaagaccg | REV 58028 bp ΦTE | - |
| TRB174 | cgtctttgaacaggaagc | FWD 74985 bp ΦTE | - |
| TRB175 | tcgctgtggtacagtacc | REV 76030 bp ΦTE | - |
| TRB176 | aacatcggctgagtcagg | FWD 76745 bp ΦTE | - |
| TRB177 | cttcaagaaatccgctgc | REV 77764 bp ΦTE | - |
| TRB178 | cctcttgtcttatctgcc | FWD 87983 bp ΦTE | - |
| TRB179 | aagagccttggtctatgc | REV 89012 bp ΦTE | - |
| TRB180 | ttatggtcaccgaacagc | FWD 92100 bp ΦTE | - |
| TRB181 | aaaacggcgcaagctatg | REV 93133 bp ΦTE | - |
| TRB182 | taatcccatctaccaccc | FWD 98777 bp ΦTE | - |
| TRB183 | ggatgatggagacatacc | REV 99813 bp ΦTE | - |
| TRB184 | gataagtgacgaacttgc | FWD 109200 bp ΦTE | - |
| TRB185 | taattcctgatgccacgg | REV 110727 bp ΦTE | - |
| TRB186 | tagacttcaccaagctgg | FWD 115077 bp ΦTE | - |
| TRB187 | cttttgttcgtcagtgcg | REV 116171 bp ΦTE | - |
| TRB188 | taaaggtgacaccggagc | FWD 118277 bp ΦTE | - |
| TRB189 | catcatagacgttaccgc | REV 119275 bp ΦTE | - |
| TRB190 | tgaacacaggtaatctgg | FWD 139881 bp ΦTE | - |
| TRB191 | ccagtcgtatcttgctcg | REV 140934 bp ΦTE | - |
| TRB192 | gctgcaaactccattacg | FWD 140993 bp ΦTE | - |
| TRB193 | tgccgacttgtaaattcc | REV 142077 bp ΦTE | - |
| TRB194 | agttctcttttgacgggc | FWD 106196 bp ΦTE | - |
| TRB195 | aagacaagctgcccttgg | REV 107232 bp ΦTE | - |
| TRB196 | aggttgccgccattgttg | FWD 141836 bp ΦTE | - |
| TRB197 | cgatgacgtgccattctg | REV 555 bp ΦTE | - |
| TRB198 | tagtgaccatcgacgtgg | FWD 104363 bp ΦTE | - |
| TRB199 | cttaaagttgggcagagg | REV 105340 bp ΦTE | - |
| TRB200 | *TTTAAGCTT*ACTTAAAGGTAGCGAATCACCTGGATTTTCGTCCCGC*TTGAATCTATTATAATTGTTATCCG* | REV ΦTE-A DNA repeat (3T) | HindIII |
| TRB201 | *TTTAAGCTT*ACTTAAGGTAGCGAATCACCTGGATTTTCGTCCCGC*TTGAATCTATTATAATTGTTATCCG* | REV ΦTE-A DNA repeat (2T) | HindIII |
| TRB202 | *TTTAAGCTT*TTTCGTCCCGCACTTAAAGGTAGCGAATCACCTGGAT*TTGAATCTATTATAATTGTTATCCG* | REV ΦTE-A RNA repeat (3T) | HindIII |
| TRB203 | *TTTAAGCTT*TTTCGTCCCGCACTTAAGGTAGCGAATCACCTGGAT*TTGAATCTATTATAATTGTTATCCG* | REV ΦTE-A RNA repeat (2T) | HindIII |
| TRB204 | *TTTAAGCTT*GGATTTTCGTCCCGCACTTAAAGGTAGCGAATCACCTTTGAATCTATTATAATTGTTATCCG | REV ΦTE-A DNA repeat (3T) matching phasing of ToxI | HindIII |
| TRB205 | *TTTAAGCTT*GGATTTTCGTCCCGCACTTAAGGTAGCGAATCACCT*TTGAATCTATTATAATTGTTATCCG* | REV ΦTE-A DNA repeat (2T) matching phasing of ToxI | HindIII |
| TRB206 | *TTTAAGCTT*ACTTAAAGGTAGCAAATCACCTGAATTTCTAGCTGC*TTGAATCTATTATAATTGTTATCCG* | REV ΦTE-F DNA repeat | HindIII |
| TRB208 | CAATTATAATAGATTCAAATTGACTCTATAGCTCAG | FWD ΦTE-F DNA repeat matching phasing of ToxI | AanI |
| TRB209 | TTAAGCTTTCGAACCTCGGACCTGCG | REV ΦTEescape locus | HindIII |
| TRB210 | CAATTATAATAGATTCAATCAGGTATACTTAAGTTA | FWD ToxI Pba SCRI1039 pECA1039 | AanI |
| TRB211 | TTAAGCTTCGCCTCTTTTACTTTATTAC | REV ToxI Pba SCRI1039 pECA1039 | HindIII |
| TRB217 | TTGTATACTTAAGTTATTGACTCTATAGCTCAG | FWD ToxI Pba SCRI1039 pECA1039 | BstZ171 |
| TRB218 | TTGACTATGTAGTCGCCTCTTTTACTTTATTTCGAACCTCGGACCTGCG | REV ToxI Pba SCRI1039 pECA1039 | DrdI |
| TRB246 | TTGAATTCACACCCTCCTTTGAAACC | REV 99536 bp ΦTE | EcoRI |
| TRB251 | TTGAATTCATCAGGTATACTTAAGTTA | FWD ToxI Pba SCRI1039 pECA1039 | EcoRI |
| TRB252 | TTGAATTCATTGACTCTATAGCTCAG | FWD ΦTE escape locus | EcoRI |
| TRB265 | TTTCTAGAGTAAGATCTATATAAGATC | FWD 99267 bp ΦTE | XbaI |
| TRB281 | *TTTAAGCTT*GAATTTCTAGCTGCACTTAAAGGTAGCAAATCACCTGAATTTCT*TTGAATCTATTATAATTGTTATCCG* | REV Single repeat ToxI altered phase to include whole pseudoknot and cutting sites (3T) | - |
| TRB282 | *TTTAAGCTT*GAATTTCTAGCTGCACTTAAGGTAGCAAATCACCTGAATTTCT*TTGAATCTATTATAATTGTTATCCG* | REV Single repeat ToxI altered phase to include whole pseudoknot and cutting sites (2T) ie. mutation 3 | - |
| TRB292 | *TTTAAGCTT*GAATTTCTAGCTGCACTTAAAGGTAGCAAATCACCTGGATTTCT*TTGAATCTATTATAATTGTTATCCG* | REV Single repeat variant ToxI altered phase to include whole pseudoknot and cutting sites (3T); mutation 1 | - |
| TRB293 | *TTTAAGCTT*GAATTTCTAGCCGCACTTAAAGGTAGCGAATCACCTGAATTTCT*TTGAATCTATTATAATTGTTATCCG* | REV Single repeat variant ToxI altered phase to include whole pseudoknot and cutting sites (3T); mutation 2 | - |
| TRB294 | *TTTAAGCTT*GAATTTCGTCCTGCACTTAAAGGTAGCAAATCACCTGAATTTCT*TTGAATCTATTATAATTGTTATCCG* | REV Single repeat variant ToxI altered phase to include whole pseudoknot and cutting sites (3T); mutation 4 | - |
| TRB295 | *TTTAAGCTT*GAATTTTCTAGCTGCACTTAAAGGTAGCAAATCACCTGAATTTCT*TTGAATCTATTATAATTGTTATCCG* | REV Single repeat variant ToxI altered phase to include whole pseudoknot and cutting sites (3T); mutation 5 | - |
| TRB297 | *TTTAAGCTT*GAATTTTCGTCCCGCACTTAAGGTAGCGAATCACCTGGATTTCT*TTGAATCTATTATAATTGTTATCCG* | REV Single repeat pseudo-ToxI altered phase to include whole pseudoknot and cutting sites (2T); ie., ToxI with mutations 1, 2, 3, 4, 5 | - |
| TRB308 | *TTTAAGCTT*GAATTTTCTAGCCGCACTTAAGGTAGCGAATCACCTGGATTTCT*TTGAATCTATTATAATTGTTATCCG* | REV Single repeat variant ToxI altered phase to include whole pseudoknot and cutting sites (2T); mutations 1, 2, 3, 5 | - |
| TRB309 | *TTTAAGCTT*GAATTTTCGAGCCGCACTTAAGGTAGCGAATCACCTGGATTTCT*TTGAATCTATTATAATTGTTATCCG* | REV Single repeat variant ToxI altered phase to include whole pseudoknot and cutting sites (2T); mutations 1, 2, 3, 5, A29C | - |
| TRB310 | *TTTAAGCTT*GAATTTTCTTGCCGCACTTAAGGTAGCGAATCACCTGGATTTCT*TTGAATCTATTATAATTGTTATCCG* | REV Single repeat variant ToxI altered phase to include whole pseudoknot and cutting sites (2T); mutations 1, 2, 3, 5, U28A | - |
| TRB311 | *TTTAAGCTT*GAATTTTCTACCCGCACTTAAGGTAGCGAATCACCTGGATTTCT*TTGAATCTATTATAATTGTTATCCG* | REV Single repeat variant ToxI altered phase to include whole pseudoknot and cutting sites (2T); mutations 1, 2, 3, 5, C27G | - |
| TRB315 | *TTTAAGCTT*GAATTTCGAGCTGCACTTAAAGGTAGCAAATCACCTGAATTTCT*TTGAATCTATTATAATTGTTATCCG* | REV Single repeat variant ToxI altered phase to include whole pseudoknot and cutting sites (3T); mutation A29C | - |
| TRB316 | *TTTAAGCTT*GAATTTCTTGCTGCACTTAAAGGTAGCAAATCACCTGAATTTCT*TTGAATCTATTATAATTGTTATCCG* | REV Single repeat variant ToxI altered phase to include whole pseudoknot and cutting sites (3T); mutation U28A | - |
| TRB317 | *TTTAAGCTT*GAATTTCTACCTGCACTTAAAGGTAGCAAATCACCTGAATTTCT*TTGAATCTATTATAATTGTTATCCG* | REV Single repeat variant ToxI altered phase to include whole pseudoknot and cutting sites (3T); mutation C27G | - |
|  |  |  |  |
|  | | | |
| **a.** Primers in lower case were used to generate amplicons for Sanger sequencing specific regions of ΦTE. Italics indicate primer bases forming part of the pTA100 vector backbone bordering the inserted antitoxic sequence. | | | |
| **b.** FWD, forward; REV, reverse. | | | |
