# Supplementary material for: Viral Evasion of a Bacterial Suicide System by RNA–Based Molecular Mimicry Enables Infectious Altruism
Source: PLoS Genet. 2012 Oct 18;8(10):e1003023. doi: 10.1371/journal.pgen.1003023 (PMC3475682; doi:10.1371/journal.pgen.1003023)
Supplement: Table S6 — Plasmids used in this study. (DOCX) [file pgen.1003023.s007.docx]

| **Table S6.** Plasmids used in this study | | | |
| --- | --- | --- | --- |
| Plasmid | Relevant characteristic(s)^a^ | Reference or Source | Primers for construction |
|  |  |  |  |
|  |  |  |  |
| pACYC184 | Cloning vector, p15A origin, Cm^R^ | [26] | - |
| pBR322 | Cloning vector, ColE1 origin, Ap^R^, Tc^R^ | New England Biolabs | - |
| pBAD30 | Expression vector, repressed by glucose, induced by L-arabinose, p15A origin, Ap^R^ | [25] | - |
| pBluescriptII KS+ | Cloning vector, ColE1 origin, *lacZα*, Ap^R^ | Fermentas | - |
| pBluescriptII SK- | Cloning vector, ColE1 origin, *lacZα*, Ap^R^ | Fermentas | - |
| pKD46 | λ red recombinase vector, Ap^R^ | [28] | - |
| pQE-80L | Expression vector, induced by IPTG, Ap^R^ | Qiagen | - |
| pECA1039-Km3 | EZ::Tn^TM^ <*Not*I/KAN-3> mutant in pECA1039 *orf5*, Km^R^, Abi+ | [11] | - |
| pECA1039-Km12 | EZ::Tn^TM^ <*Not*I/KAN-3> mutant in pECA1039 OriV locus, Km^R^, Abi+ | [11] | - |
| pECA1039-Km23 | EZ::Tn^TM^ <*Not*I/KAN-3> mutant in pECA1039 *toxN*, Km^R^, Abi- | [11] | - |
| pFLS50 | *In vitro* transcription vector for *E. coli ompA*, pBluescriptII KS+, ApR | Francesca Short, gifted plasmid | - |
| pFLS66 | *In vitro* transcription vector for ToxI from *Bacillus thuringiensis*, pBluescriptII KS+, ApR | Francesca Short, gifted plasmid | - |
| pMJ4 | ToxIN, with ToxN containing a C-terminal FLAG tag, cloned into pBR322, Ap^R^ | [12] | - |
| pNRW124 | Plasmid for transposon mutagenesis, Km^R^ transposon | Neil Williamson, gifted plasmid | - |
| pTA46 | ToxIN cloned into pBR322, Ap^R^ | [11] | - |
| pTA47 | ToxIN cloned into pBR322, with frame-shift mutation in *toxN*, referred to as 'ToxIN-FS', Ap^R^ | [11] | - |
| pTA49 | Inducible ToxN cloned into pBAD30, Ap^R^ | [12] | - |
| pTA96 | ToxIN cloned into pBR322, with frame-shift mutation in *toxN*, and *toxI* only containing 1.5 repeats, Ap^R^ | This study | See Materials and Methods |
| pTA100 | pQE-80L derivative, IPTG-inducible promoter, ColE1 origin, Sp^R^, Sm^R^ | [11] | - |
| pTA103 | ToxI from pECA1039 single repeat expression vector, 'ToxI DNA repeat (3T)', pTA100-based, Sp^R^, Sm^R^ | [11] | - |
| pTA110 | *In vitro* transcription vector for antisense ToxI RNA, pBluescriptII SK-, Ap^R^ | This study | TRB57, MJ12 |
| pTRB1 | ToxN containing a C-terminal FLAG tag, cloned into pBAD30, Ap^R^ | [11] | - |
| pTRB101 | ToxIN from pTA46 cloned into pACYC184, Cm^R^ | This study | KD01, TRB63 |
| pTRB102 | ToxIN-FS from pTA46 cloned into pACYC184, Cm^R^ | This study | KD01, TRB63 |
| pTRB165 | ΦTE-F DNA repeat cloned into pTA100, amplified using pQE-80L as template, Sp^R^, Sm^R^ | This study | PF185, TRB206 |
| pTRB169 | ΦTE-F escape locus cloned into pTA100, Sp^R^, Sm^R^ | This study | TRB208, TRB209 |
| pTRB170 | ΦTE-F DNA repeat matching phasing of ToxI, cloned into pTA100, Sp^R^, Sm^R^ | This study | TRB208, TRB209 |
| pTRB171 | ΦTE wild type escape locus cloned into pTA100, Sp^R^, Sm^R^ | This study | TRB208, TRB209 |
| pTRB172 | ΦTE-A escape locus cloned into pTA100, Sp^R^, Sm^R^ | This study | TRB208, TRB209 |
| pTRB175 | ToxI cloned into pTA100, Sp^R^, Sm^R^ | This study | TRB210, TRB211 |
| pTRB193 | ΦTE-F escape locus cloned into pECA1039-Km3, Km^R^ | This study | TRB217, TRB218 |
| pTRB200 | ΦTE-A DNA repeat (3T) cloned into pTA100, amplified using pQE-80L as template, Sp^R^, Sm^R^ | This study | PF185, TRB200 |
| pTRB201 | ΦTE-A DNA repeat (2T) cloned into pTA100, amplified using pQE-80L as template, Sp^R^, Sm^R^ | This study | PF185, TRB201 |
| pTRB202 | ΦTE-A RNA repeat (3T) cloned into pTA100, amplified using pQE-80L as template, Sp^R^, Sm^R^ | This study | PF185, TRB202 |
| pTRB203 | ΦTE-A RNA repeat (2T) cloned into pTA100, amplified using pQE-80L as template, Sp^R^, Sm^R^ | This study | PF185, TRB203 |
| pTRB204 | ΦTE-A DNA repeat (3T) matching phasing of ToxI, cloned into pTA100, amplified using pQE-80L as template, Sp^R^, Sm^R^ | This study | PF185, TRB204 |
| pTRB205 | ΦTE-A DNA repeat (2T) matching phasing of ToxI, cloned into pTA100, amplified using pQE-80L as template, Sp^R^, Sm^R^ | This study | PF185, TRB205 |
| pTRB258 | ΦTE wild type genomic region cloned into pBluescriptII SK-, Ap^R^ | This study | TRB265, TRB246 |
| pTRB259 | ToxI cloned into pBluescriptII SK-, Ap^R^ | This study | TRB251, TRB211 |
| pTRB260 | ΦTE wild type escape locus cloned into pBluescriptII SK-, Ap^R^ | This study | TRB252, TRB209 |
| pTRB261 | ΦTE-F escape locus cloned into pBluescriptII SK-, Ap^R^ | This study | TRB252, TRB209 |
| pTRB262 | ΦTE-A escape locus cloned into pBluescriptII SK-, Ap^R^ | This study | TRB252, TRB209 |
| pTRB263 | *E. coli ompA* cloned into pBluescriptII SK-, SacI-HindIII fragment from pFLS50, Ap^R^ | This study | - |
| pTRB264 | ToxI from *Bacillus thuringiensis* cloned into pBluescriptII SK-, BamHI-HindIII fragment from pFLS66, Ap^R^ | This study | - |
| pTRB266 | ToxI wild type (3T) cloned into pTA100, amplified using pQE-80L as template, Sp^R^, Sm^R^ | This study | PF185, TRB281 |
| pTRB267 | ToxI variant (2T) cloned into pTA100, amplified using pQE-80L as template, Sp^R^, Sm^R^, carrying mutation 3 | This study | PF185, TRB282 |
| pTRB272 | ToxI variant (3T) cloned into pTA100, amplified using pQE-80L as template, Sp^R^, Sm^R^, carrying mutation 1 | This study | PF185, TRB292 |
| pTRB273 | ToxI variant (3T) cloned into pTA100, amplified using pQE-80L as template, Sp^R^, Sm^R^, carrying mutation 2 | This study | PF185, TRB293 |
| pTRB274 | ToxI variant (3T) cloned into pTA100, amplified using pQE-80L as template, Sp^R^, Sm^R^, carrying mutation 4 | This study | PF185, TRB294 |
| pTRB275 | ToxI variant (3T) cloned into pTA100, amplified using pQE-80L as template, Sp^R^, Sm^R^, carrying mutation 5 | This study | PF185, TRB295 |
| pTRB288 | ToxI variant (2T) cloned into pTA100, amplified using pQE-80L as template, Sp^R^, Sm^R^, carrying mutations 1, 2, 3, 5 | This study | PF185, TRB308 |
| pTRB289 | pseudo-ToxI (2T) cloned into pTA100, amplified using pQE-80L as template, Sp^R^, Sm^R^, ie. ToxI carrying all mutations 1, 2, 3, 4, 5 | This study | PF185, TRB297 |
| pTRB290 | ToxI variant (2T) cloned into pTA100, amplified using pQE-80L as template, Sp^R^, Sm^R^, carrying mutations 1, 2, 3, 5, A29C | This study | PF185, TRB309 |
| pTRB291 | ToxI variant (2T) cloned into pTA100, amplified using pQE-80L as template, Sp^R^, Sm^R^, carrying mutations 1, 2, 3, 5, U28A | This study | PF185, TRB310 |
| pTRB292 | ToxI variant (2T) cloned into pTA100, amplified using pQE-80L as template, Sp^R^, Sm^R^, carrying mutations 1, 2, 3, 5, C27G | This study | PF185, TRB311 |
| pTRB296 | ToxI variant (3T) cloned into pTA100, amplified using pQE-80L as template, Sp^R^, Sm^R^, carrying mutation A29C | This study | PF185, TRB315 |
| pTRB297 | ToxI variant (3T) cloned into pTA100, amplified using pQE-80L as template, Sp^R^, Sm^R^, carrying mutation U28A | This study | PF185, TRB316 |
| pTRB298 | ToxI variant (3T) cloned into pTA100, amplified using pQE-80L as template, Sp^R^, Sm^R^, carrying mutation C27G | This study | PF185, TRB317 |
|  |  |  |  |
|  | | | |
| **a.** Ap^R^, ampicillin resistance; Km^R^, kanamycin resistance; Tc^R^, tetracycline resistance; Sp^R^, Spectinomycin resistance; Sm^R^, streptomycin resistance | | | |
