# Supplementary material for: Viral Evasion of a Bacterial Suicide System by RNA–Based Molecular Mimicry Enables Infectious Altruism
Source: PLoS Genet. 2012 Oct 18;8(10):e1003023. doi: 10.1371/journal.pgen.1003023 (PMC3475682; doi:10.1371/journal.pgen.1003023)
Supplement: Text S1 — Contains further Results, Materials and Methods, and References. (DOCX) [file pgen.1003023.s008.docx]

**Supporting Text**

**Viral evasion of a bacterial suicide system by RNA-based molecular mimicry enables infectious altruism**

Tim R. Blower^1^, Terry J. Evans^1^, Rita Przybilski^2^, Peter C. Fineran^2^, George P. C. Salmond^1^

^1^Department of Biochemistry, University of Cambridge, Cambridge, CB2 1QW, United Kingdom; ^2^Department of Microbiology and Immunology, University of Otago, P.O. Box 56, Dunedin 9054, New Zealand.

Correspondence should be addressed to George P.C. Salmond at Department of Biochemistry, University of Cambridge, Cambridge, CB2 1QW, United Kingdom. Email: gpcs@mole.bio.cam.ac.uk or gpcs2@cam.ac.uk. Tel. +44(0)1223 333650.

**Supporting Results**

**Characterisation of ΦTE.** Transposon mutagenesis identified ΦTE-resistant strains of Pba that were mutant in flagellum biosynthesis genes, suggested that the flagellum may act as receptor for this phage (Table S1). The genome of ΦTE was then extracted and analysed by restriction enzyme digestion, together with genomic DNA from phages ΦAT1 and ΦM1, used as controls. These analyses indicated that the genome was not heavily modified and that there were no *cos* sites present in the genome (Figure S1A). This suggested that the ΦTE genome circularises upon injection into the host cell through homologous recombination of terminally redundant genomic ends. Following a time course of exonuclease treatment with Bal-31, which degrades dsDNA from both ends, followed by restriction enzyme digestion, specific restriction fragments were lost from the genomic digests of ΦAT1 and ΦM1 (Figure S1B), suggesting these two phages are not circularly permuted, whilst all restriction fragments from ΦTE were degraded (Figure S1C). Together, these data indicate that the linear genome of ΦTE is both terminally redundant and circularly permuted.

**ΦTE genome analysis.** ΦTE was sequenced as a single lane of the Roche 454 sequencer, returning a single raw contig of 142,353 bp with 35× coverage. ΦTE-A, ΦTE-C and ΦTE-E were made into three libraries, pooled and sequenced in a second lane. This returned single raw contigs for each phage, of 142,322 bp, 142,314 bp and 142,294 bp, respectively, with coverage at 21.1×, 38× and 24× for each. Following direct *in silico* comparison of the returned escape phage genome sequences against the wt sequence, 18 independent sites of single base-pair additions or deletions were identified, nine of which were observed in all three escape phages. The escape sequences also suggested that each escape phage had undergone a chromosomal deletion and re-arrangement, centred around 106,700 bp into the wt scaffold. Specific primer pairs TRB164-199 (Table S5) were used to finish the sequence at the putatively mutated regions, which showed that only the expansion at 106,700 bp was a true mutation, the rest were sequencing artifacts.

The genome of ΦTE is split into four gene clusters. GC skew, representing strand-specific mutational bias and which corresponds well with ‘strandedness’ of coding sequences [47] supports the proposed arrangement of the gene clusters (Figure 1B). Gene cluster 1 contains genes involved in DNA replication, such as DNA polymerase and primase. Gene cluster 2 contains few genes with clearly identifiable functionality, though there are multiple conserved hypothetical proteins. The genes present in cluster 3 predominantly represent DNA repair and nucleotide metabolism functions. Gene cluster 3 also contains the endolysin (phiTE_147) though no holin was identified. The fourth gene cluster begins with some metabolism genes and then encodes the structural components of the virion. The ΦTE terminase appears to be encoded by genes phiTE_206 and phiTE_208, suggesting that splicing is necessary for production of the active protein. The arrangement of the gene clusters suggests that at least four promoters drive transcription of this phage, though it was not possible to accurately predict the positions of these promoters using BPROM (Softberry, Inc.).

One ΦTE tRNA gene has the anti‐codon ‘GCA’. Recognition of the TGC codon would incorporate cysteine into a growing polypeptide chain. Since the presence of tRNA genes in phage genomes can be indicative of extraordinary codon usage, the relative synonymous codon usage scores (RSCU) were determined for each codon, both in Pba and ΦTE. The RSCU score for the TGC codon in ΦTE is marginally higher than in Pba (1.157 compared to 1.057) and this does not seem to justify the presence of this tRNA in ΦTE. Two of the three ΦTE genes making heaviest use of the TGC codon (phiTE_152 and phiTE_145 – ten and eight uses, respectively) are involved in the synthesis of dNTPs, and thus it is seductive to suggest that the presence of this tRNA ensures high‐level production of dNTPs for DNA replication. The second tRNA, for tyrosine, has a GTA anticodon. The complementary TAC codon is used more frequently than would be expected in ΦTE, assuming no bias, (RSCU of 1.198), but is under‐used in Pba (RSCU of 0.812), which may account for the presence of this second tRNA in the genome of ΦTE.

**Generalised transduction with ΦTE.** Whilst performing transductions with ΦTE, it was noted that it was not possible to transduce some markers, such as that of strain SCC27 [48] or plasmid pBR322 (NEB). Though at least three attempts were made for each, no transductants were recovered. In these cases, the efficiency is either below detectable levels, or there is some contextual problem preventing certain DNA sequences or replicons from being transduced. Transduction of pTRB101 by ΦTE wt required an initial step of plating ~10^7^ phages more than required for the escape phages ΦTE-A and -F. Taking into account this numerical difference, the relative transduction efficiency for a ΦTE wt phage to transfer pTRB101 is the product of the initial EOP (1 x 10^-7^) multiplied by the observed transduction efficiency (~1 x 10^-7^), making ~1 x 10^-14^. This level of efficiency would be undetectable under regular lab conditions. The evolution of ΦTE escape phages allowed transduction of pTRB101 to become as efficient as for other, non-ToxIN, plasmids and chromosomal markers (Table 1).

**Supporting Materials and Methods**

**Phage genomic DNA digestion.** Phage genomic DNAs were prepared as per the main article *Materials and Methods* section. All nucleases were purchased from New England Biolabs. Restriction digests of phage DNA contained ~500 ng DNA and were performed as directed by the manufacturer, using 2 µl enzyme for 16 hr, or as time indicated for Bal-31. DNA was visualised on ethidium bromide‐stained 1% agarose gels with a Gene Genius BioImaging System (Syngene).

**Transposon mutagenesis and identification of insertion sites.** Equal volumes of overnight cultures of Pba and β2163 (pNRW124) [49] were mixed, then 30 µl aliquots were spotted on LBA plates supplemented with 300 µM diaminopimelic acid. These mating patches were incubated for 8 hr at 25°C, then resuspended in 1 ml LB. Transposon mutants that were resistant to ΦTE were selected by mixing 200 µl of the mating suspension with 200 µl of ΦTE lysate and 3 ml of top-LBA, which was poured onto an LBA plate. Bacteriophage-resistant colonies were picked after two days of incubation at 25°C and streaked twice to remove any remaining bacteriophage. The positions of the transposon insertions were determined using a two-round random-primed PCR approach [44,50].

**Supporting Information Additional References**

All references within *Supporting Information* are included within the main reference section, with the exception of those below:

47. Marin A, Xia X (2008) GC skew in protein-coding genes between the leading and lagging strands in bacterial genomes: new substitution models incorporating strand bias. J Theor Biol 253:508-513.

48. Coulthurst SJ, Lilley KS, Hedley PE, Liu H, Toth IK, et al. (2008) DsbA plays a critical and multifaceted role in the production of secreted virulence factors by the phytopathogen *Erwinia carotovora* subsp. *atroseptica*. J Biol Chem 283:23739-23753.

49. Demarre G, Guerout AM, Matsumoto-Mashimo C, Rowe-Magnus DA, Marliere P, et al. (2005) A new family of mobilizable suicide plasmids based on broad host range R388 plasmid (IncW) and RP4 plasmid (IncPα) conjugative machineries and their cognate *Escherichia coli* host strains. Res Microbiol 156:245-255.

50. Jacobs MA, Alwood A, Thaipisuttikul I, Spencer D, Haugen E, et al. (2003) Comprehensive transposon mutant library of *Pseudomonas aeruginosa*. Proc Natl Acad Sci U S A 100:14339-14344.
